# Pharmacological and Molecular Docking Investigation of Leaves of *Eriobotrya japonica*: Antioxidant, Enzyme Inhibition, and Anti-Inflammatory Effects

**DOI:** 10.3390/antiox14040413

**Published:** 2025-03-29

**Authors:** Pao-Jen Kuo, Li-Ting Chen, Sin-Min Li, Zih-Rong Chen, Jih-Jung Chen

**Affiliations:** 1 Department of Plastic Surgery, Kaohsiung Chang Gung Memorial Hospital, Kaohsiung 833401, Taiwan; bow110470@gmail.com; 2 College of Medicine, Chang Gung University, Taoyuan 333323, Taiwan; 3 Department of Pharmacy, School of Pharmaceutical Sciences, National Yang Ming Chiao Tung University, Taipei 112304, Taiwan; linting0121@gmail.com (L.-T.C.); samuel147samuel147@gmail.com (S.-M.L.); jjasmine81023@gmail.com (Z.-R.C.); 4 Department of Medical Research, China Medical University Hospital, China Medical University, Taichung 404333, Taiwan; 5 Traditional Herbal Medicine Research Center, Taipei Medical University Hospital, Taipei 110301, Taiwan

**Keywords:** *Eriobotrya japonica*, antioxidant, anti-inflammation, anti-α-glucosidase, anti-acetylcholinesterase, molecular docking

## Abstract

Leaves of *Eriobotrya japonica* have long been utilized in traditional Chinese medicine (TCM) for treating pulmonary inflammation and stomach disorders. This study extends their pharmacological applications by evaluating the antioxidant, anti-α-glucosidase, anti-acetylcholinesterase (AChE), and anti-inflammatory activities of solvent extracts and isolated bioactive components through an integrative approach combining extraction, bioassays, and molecular docking. Solvent extracts prepared with varying polarities exhibited distinct bioactivities, with the 100 °C water and methanol extracts displaying the strongest antioxidant potential. The ethyl acetate extract exhibited potent α-glucosidase inhibition, whereas the *n*-hexane extract demonstrated significant AChE inhibitory activity. Among the isolated compounds, epicatechin (**5**) (SC_50_ = 7.83 ± 0.34 μM) and rutin (**6**) (SC_50_ = 6.69 ± 0.25 μM) showed superior ABTS and superoxide scavenging activities, respectively, compared to the positive controls (BHT and cynaroside). Ursolic acid (**2**) exhibited stronger α-glucosidase inhibition (IC_50_ = 10.68 ± 0.76 μM) than acarbose (IC_50_ = 419.93 ± 29.15 μM), while tormentic acid (**4**) demonstrated superior AChE inhibition compared to chlorogenic acid. Ursolic acid (**2**) also displayed NO inhibition (IC_50_ = 20.18 ± 1.46 μM) comparable to quercetin (IC_50_ = 17.05 ± 1.63 μM), with Western blot analysis confirming its potent iNOS inhibitory activity. Molecular docking further supported these findings, revealing that ursolic acid (**2**) exhibited stronger binding affinity to α-glucosidase (−8.7 kcal/mol) than acarbose (−5.1 kcal/mol), tormentic acid (**4**) displayed higher binding energy to AChE (−8.8 kcal/mol) compared to chlorogenic acid (−7.8 kcal/mol), and ursolic acid (**2**) (−7.5 kcal/mol) showed a binding affinity to iNOS similar to that of quercetin (−7.7 kcal/mol). These results highlight the strong potential of *E. japonica* leaf extracts and bioactive compounds as natural antioxidants, enzyme inhibitors, and anti-inflammatory agents, supporting their development as dietary supplements or therapeutic candidates for managing oxidative stress, hyperglycemia, neurodegenerative diseases, and inflammatory disorders.

## 1. Introduction

Oxidative stress is a critical biological process that is caused by an imbalance in the production of oxygen reactive species (ROS), such as superoxide anion (O_2_^•−^), hydroxyl radical (^•^OH), and hydroperoxyl radical (HOO^•^) [1]. When the production of ROS exceeds the defense capacity, oxidative stress will result in damage to DNA, proteins, and cellular membranes [2]. Moreover, many diseases, including cancer, cardiovascular disorders, neurodegenerative diseases, and diabetes, are associated with the imbalance of oxidative stress [3]. In the effort to reduce oxidative stress and its associated health risks, the exploration of natural antioxidants has gained significant attention.

Type 2 diabetes (T2DM) is a chronic metabolic disorder, characterized by insulin resistance and hyperglycemia, which can result in severe complications, such as cardiovascular disease, kidney failure, and neuropathy [4]. Postprandial hyperglycemia is a critical factor in preventing long-term complications and can be effectively controlled by α-glucosidase inhibitors [5]. However, the most clinically available α-glucosidase inhibitors are synthetic and frequently associated with gastrointestinal adverse effects, including diarrhea, nausea, and flatulence [6]. As a result, traditional Chinese herbal medicines with low levels of side effects have gained attention as promising sources of therapy for natural anti-α-glucosidase agents.

Alzheimer’s disease (AD) is a worldwide prevalent neurodegenerative disorder that affects millions of people [7]. It results in significant reductions in acetylcholine (ACh) levels in brain regions critical for learning and memory and is characterized by the progressive degeneration of cholinergic neurons [8]. Mitigating the breakdown of ACh by acetylcholinesterase (AChE) inhibitors is one of the therapeutic strategies employed, thereby enhancing its level and prolonging its action as a neurotransmitter [9]. However, commonly used synthetic AChE inhibitors, such as donepezil, rivastigmine, and galantamine, often lead to many adverse effects, including loss of appetite, nausea, vomiting, and diarrhea, which limits their suitability for long-term use [10]. Therefore, natural products derived from medicinal herbs have gained attention as promising candidates for the development of safer and more tolerable therapies for Alzheimer’s disease.

Inflammation response and its associated disorders are crucial recent issues in medicine, presenting considerable challenges for healthcare providers and researchers [11]. Inflammation is a process driven by mediating pro-inflammatory cytokines in response to allergens, injury, or infection [12]. It leads to the accumulation of leukocytes and results in continuous stimuli and tissue damage [13]. In clinical settings, inflammation can be classified into acute and chronic inflammation [13]. Acute inflammation is a rapid response to bacterial infection or tissue damage [14], while chronic inflammation occurs when the body fails to eliminate pathogens or when the immune system becomes hyperactivated [15], leading to prolonged inflammation, such as in rheumatoid arthritis [16], lupus erythematosus [17], and Crohn’s disease [18]. Therefore, the development of potent anti-inflammatory agents for the management and prevention of inflammatory diseases remains a critical area of research.

*Eriobotrya japonica* Lindl.*,* commonly known as loquat, is a subtropical evergreen fruit tree of economic significance. It is native to southeastern China and belongs to the Rosaceae plants [19]. *E. japonica* is not only valued for its fruit, with rich nutrients and known for its antioxidant and anti-inflammatory properties [19,20,21], but also for its seeds and flowers, which are used in traditional medicine for their potential analgesic, anti-tumor, and respiratory health benefits [22]. Among its various parts, the leaves have gained particular attention due to their extensive use in TCM for treating chronic bronchitis, cough, nausea, and other ailments, and their pharmacological activities, including antioxidant [23], anti-inflammatory [24,25], and antidiabetic effects [26,27]. Several important bioactive components have been found in the leaves of *E. japonica*, including triterpenes [26], sesquiterpenes [28], flavonoids [26], tannins, and megastigmane glycosides [29]. Among these, triterpenoids, such as corosolic acid, ursolic acid, tormentic acid, oleanolic acid, and maslinic acid [30], and flavonoids, such as epicatechin, rutin, procyanidin C1, and naringenin [31], are the most pharmacologically active compounds [26,32]. On the other hand, strong antioxidant activities are also found in the leaves of *E. japonica* [33]. Additionally, the reduction of free radicals and increased consumption of antioxidant-rich foods or supplements are known to mitigate the risk of free radical-induced diseases [34]. In recent years, natural products have attracted growing attention as promising sources of safer and more effective therapeutic alternatives [35]. In our past research, many potent bioactive components from natural sources have been discovered [36,37,38,39,40,41]. Although several components from *E. japonica* have been identified, its biological activities remain underexplored. Therefore, this study explored the pharmacological potential of various extracts from different solvents and major components from leaves of *E. japonica*, focusing on their antioxidant, anti-α-glucosidase, and anti-acetylcholinesterase activities. Furthermore, molecular docking analyses were conducted to investigate the interactions between major bioactive compounds and enzyme targets.

## 2. Materials and Methods

### 2.1. Chemicals and Reagent

All chemicals and reagents used in this study were analytical grade. 2,2-Diphenyl-1-(2,4,6-trinitrophenyl) hydroxyl (DPPH), nitro blue tetrazolium (NBT), and phenazine methosulphate (PMS) were purchased from Tokyo Chemical Industry Co., Ltd. (Tokyo, Japan). Chemicals from Sigma-Aldrich (St. Louis, MO, USA) included bovine serum albumin, 2,2′-azino-bis(3-ethylbenzothiazoline-6-sulfonic acid) (ABTS), Folin-Ciocalteu’s reagent, DTNB, TPTZ, EDTA, α-glucosidase, acetylcholinesterase, acetylcholine iodide, and DMSO. NADH was obtained from Acros Organics (Geel, Belgium), while ferric chloride, aluminum chloride, and *p*-NPG were sourced from Alfa Aesar (Lancashire, UK). Additional reagents such as glycine (J.T. Baker, Phillipsburg, NJ, USA), and TEMED and ammonium persulfate (Bio-Rad, Hercules, CA, USA) were also used. Disodium hydrogen phosphate, sodium carbonate, and related phosphates were supplied by SHOWA Chemical Co. Ltd. (Chuo-ku, Japan). The solvents used were *n*-hexane (ACS grade), ethyl acetate (EtOAc, ACS grade), acetone (ACS grade), 95% ethanol (EtOH, ACS grade), and methanol (MeOH, ACS grade); they were purchased from Sigma-Aldrich (St. Louis, MO, USA). The standards of acarbose and BHT were obtained from Acros Organics (Geel, Belgium) with purity ≥ 99%; quercetin, Trolox, chlorogenic acid, and gallic acid were obtained from Sigma-Aldrich (St. Louis, MO, USA) with purity ≥ 99%; cynaroside was obtained from MedChemExpress (MCE, Monmouth Junction, NJ, USA) with purity ≥ 99%.

### 2.2. Preparation of Solvent Extracts

The leaves of *E. japonica* were purchased from the Wan-An Chinese medicine shop located in Wanhua Dist., Taipei City, Taiwan, in March 2024, and identified by Prof. J.-J. Chen. The dried leaves of loquat (*E. japonica*) were sourced from Zhejiang, China, and a voucher specimen was deposited in the Department of Pharmacy, National Yang Ming Chiao Tung University, Taipei, Taiwan. The leaves of *E. japonica* were crushed and divided into six portions (25 g each) for extraction with 100 mL of deionized water, methanol, ethanol, ethyl acetate, acetone, or *n*-hexane. Each portion was soaked in a flask for 24 h, with sonication for 1 h, and the procedure was repeated three times. For the 100 °C water extract, 40 g of dried leaves of *E. japonica* were soaked in deionized water (1 L) for 30 min; this was followed by boiling for an additional 30 min until the volume was reduced by half. All extracts were filtered, and the organic solvent extracts were concentrated at 38 °C under reduced pressure, while water extracts (including the 100 °C water extract) were freeze-dried.

### 2.3. Isolation of Pure Components

**Figure 1:**
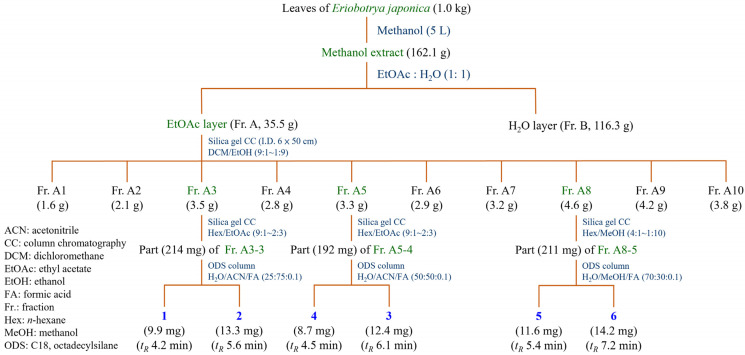
Extraction and isolation of bioactive components from *E. japonica*.

**Figure 2:**
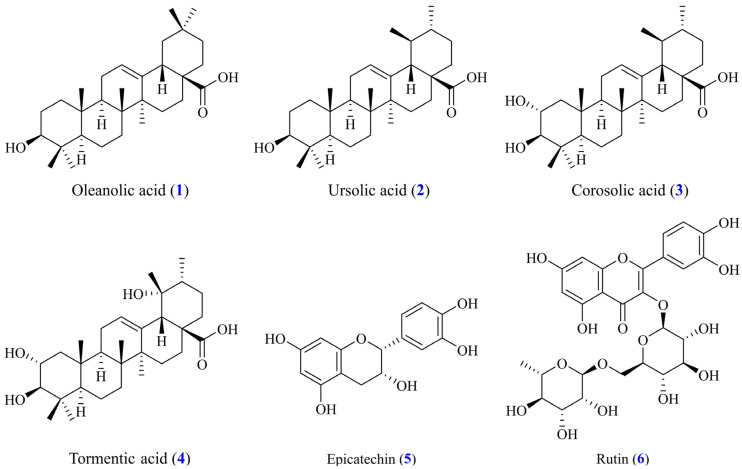
Chemical structures of oleanolic acid (**1**), ursolic acid (**2**), corosolic acid (**3**), tormentic acid (**4**), epicatechin (**5**), and rutin (**6**) from leaves of *E. japonica*.

The leaves of *Eriobotrya japonica* (1.0 kg) were soaked in 5 L of 100% methanol for 72 h at room temperature, with intermittent stirring. The extracted solution was filtered and concentrated at 38 °C under reduced pressure to obtain the dried crude extract (162.1 g) (Figure 1). The crude methanol extract was resuspended in water and sequentially partitioned with EtOAc (ethyl acetate) to obtain two fractions, including EtOAc fraction (Fr. A, 35.5 g) and water fraction (Fr. B, 116.3 g). Fr. A (35.5 g) was further separated by silica gel column chromatography (I.D. 6 × 50 cm; CH_2_Cl_2_/EtOH gradient) to afford 10 subfractions (Fr. A1–A10). The Fr. A3 (3.5 g) was subjected to silica gel column chromatography (*n*-hexane/EtOAc 9:1–2:3) to give 6 fractions (Fr. A3-1–A3-6). The reversed-phase HPLC separation system was used for further purification using a mobile phase consisting of 0.1% formic acid (FA) aqueous solution (H_2_O) (*v/v*) and acetonitrile (ACN). Part (164 mg) of Fr. A3-3 was further purified by ODS column (H_2_O/ACN/FA 25:75:0.1) to obtain compounds **1** (17.9 mg, *t_R_* = 4.2 min) and **2** (24.3 mg, *t_R_* = 5.6 min). The Fr. A5 (3.3 g) was subjected to silica gel column chromatography (*n*-hexane/EtOAc 9:1–2:3) to give 6 fractions (Fr. A5-1–A5-6). Part (142 mg) of Fr. A5-4 was further purified by ODS column (H_2_O/ACN/FA 50:50:0.1) to obtain compounds **4** (12.7 mg, *t_R_* = 4.5 min) and **3** (20.4 mg, *t_R_* = 6.1 min). The Fr. A8 (4.6 g) was subjected to silica gel column chromatography (*n*-hexane/MeOH 4:1–1:10) to give 6 fractions (Fr. A8-1–A8-6). Part (181 mg) of Fr. A8-5 was further purified by ODS column (H_2_O/ACN/FA 70:30:0.1) to obtain compounds **5** (15.6 mg, *t_R_* = 5.4 min) and **6** (18.2 mg, *t_R_* = 7.2 min). The isolated compounds, including oleanolic acid (**1**) [42], ursolic acid (**2**) [42], corosolic acid (**3**) [43], tormentic acid (**4**) [44], epicatechin (**5**) [45], and rutin (**6**) [31,46], were identified by NMR (Figures S1, S3, S5, S7, S9, and S11), and the structures are shown in Figure 2.

### 2.4. Total Phenolic Content (TPC) Determination

The Folin–Ciocalteu method was utilized to evaluate the TPC of each solvent extract [47]. Extract samples were diluted with deionized water to a concentration of 100 μg/mL. Gallic acid was used to construct a standard curve. Each diluted extract sample or gallic acid standard solution (200 μL) was mixed with Folin–Ciocalteu reagent (200 μL, 0.5 N, diluted with deionized water); this was followed by the addition of 400 μL of 20% Na_2_CO_3_ solution. The mixtures were incubated for 40 min in the dark, and the absorbance at 750 nm was measured using an ELISA reader. TPC was calculated based on the gallic acid standard curve and expressed as mg of gallic acid equivalent (GAE) per g of extract.

### 2.5. Total Flavonoid Content (TFC) Determination

The AlCl_3_ colorimetric method was used to determine the TFC of each solvent extract [47]. Extract samples were diluted with methanol to 100 μg/mL. Quercetin standard solution was used to construct a standard curve. The diluted extract sample or quercetin standard solution (200 μL) was mixed with 10% AlCl_3_ solution (100 μL) and 0.1 mM CH_3_COOK solution (100 μL). The mixtures were incubated for 30 min at room temperature, and their absorbance was measured at 415 nm. TFC was calculated based on the quercetin standard curve and expressed as mg quercetin equivalents (QE) per g of extract.

### 2.6. Radical Scavenging Activity of DPPH

According to the method reported in [48], a DPPH radical solution (400 μM) was prepared in EtOH. The tested samples were diluted to the desired concentrations with EtOH. In a 96-well plate, diluted tested sample solution (100 μL) was mixed with 100 μL of the DPPH radical solution (200 μM). The mixture was allowed to react at room temperature for 30 min in the dark. After the reaction, the absorbance at 520 nm was measured using an ELISA reader (TECAN Sunrise, Männedorf, Schweiz). The scavenging activity was calculated using the following equation.

Scavenging rate (%) = (A_control_ − A_sample_)/A_control_ × 100%

where A_control_ was the absorbance of the control and A_sample_ was that of the tested sample.

### 2.7. Radical Scavenging Activity of ABTS

According to the method reported in [49], ABTS solution (28 mM) was mixed with potassium permanganate solution (9.6 mM) in an equal ratio (*v/v* = 1:1) and allowed to react at room temperature in the dark for 16 h to generate the ABTS radical solution. The ABTS radical solution was then diluted with EtOH until the absorbance reached 0.70 ± 0.02 at 740 nm. The tested samples were subsequently diluted to the desired concentrations with EtOH. In a 96-well plate, the ABTS radical solution (190 μL) was mixed with the sample solution (10 μL) and was allowed to react at room temperature for 6 min in the dark. The absorbance was then measured at 740 nm using an ELISA reader. The scavenging activity was calculated using the following equation.

Scavenging rate (%) = (A_control_ − A_sample_)/A_control_ × 100%

where A_control_ was the absorbance of the control and A_sample_ was that of the tested sample.

### 2.8. Radical Scavenging Activity of Superoxide

According to a previously reported procedure [48], the superoxide radical solution was prepared in Tris-HCl buffer (16 mM, pH 8.0) containing 300 µM NBT (50 µL), 120 µM PMS (50 µL), and tested sample solution (50 µL). The reaction was initiated by adding 468 µM NADH solution (50 µL) and incubated at room temperature for 5 min. The absorbance at 560 nm was measured using an ELISA reader, and the scavenging activity was calculated by the following equation.

Scavenging rate (%) = (A_control_ − A_sample_)/A_control_ × 100%

where A_control_ was the absorbance of the control and A_sample_ was that of the tested sample.

### 2.9. Ferric Reducing Antioxidant Power (FRAP)

According to the previously published methods [50], acetate buffer (pH 3.6), FeCl_3_ solution (20 mM), and 10 m TPTZ solution (prepared in 40 mM HCl) were mixed in a ratio of 10:1:1 to generate the FRAP working solution. Then, the working solution was warmed to 37 °C before being used. The diluted sample, blank, or standard solution (100 µL) was mixed with the warm-up working solution (900 µL) and incubated for 40 min in a dry bath at 37 °C. The absorbance at 593 nm was measured using an ELISA reader. The standard curve was linear from 0 to 100 mM Trolox, and the data were expressed as mM TE/g dry weight.

### 2.10. Enzyme Inhibition Assay of α-Glucosidase

The inhibitory activity of α-glucosidase was determined based on the previously reported method [51]. The α-glucosidase solution was prepared with sodium phosphate buffer (pH 6.8, 0.1 M) and diluted to 1 U/mL. Subsequently, the tested sample solution (100 µL), diluted α-glucosidase solution (20 µL), and 0.53 mM *p*-NPG (380 µL) were mixed and incubated at 37 °C for 40 min. The reaction was stopped by adding Na_2_CO_3_ solution (500 μL, 0.1 M), and the absorbance was measured at 405 nm. The activity was calculated by the following equation.

α-Glucosidase inhibition (%) = (A_control_ − A_sample_)/A_control_ × 100%

where A_sample_ and A_control_ represent the absorbance values of the tested sample and control, respectively.

### 2.11. Enzyme Inhibition Assay of Acetylcholinesterase (AChE)

The AChE inhibition was carried out based on the previous method [52]. The sodium phosphate buffer (pH 8.0, 0.1 M, 140 μL), DTNB (10 μL), tested sample (20 μL), and AChE solution (15 μL) were mixed in a 96-well plate. After incubating at room temperature for 10 min, the absorbance was measured at 405 nm. The inhibitory effect was calculated by the following equation.

Acetylcholinesterase inhibition (%) = (A_control_ − A_sample_)/A_control_ × 100%

where A_sample_ and A_control_ represent the absorbance values of the tested sample and control, respectively.

### 2.12. Cell Culture

Murine macrophage RAW264.7 cells were cultured in 5% CO_2_ and a humidified atmosphere at 37 °C. The medium for the RAW264.7 cells was Dulbecco’s Modified Eagle Medium containing 10% fetal bovine serum (FBS) and 1% penicillin. Cells were subcultured every 2 days, keeping the cell density between 1 × 10^5^ and 2 × 10^6^ cells/mL.

### 2.13. Cell Viability Assay

Cell viability of the RAW264.7 cells was determined by MTT assay [53]. A 100 μL quantity of the cells was seeded in a 96-well plate at a density of 5 × 10^5^ cells/mL and incubated for 24 h. The cells were then treated with different concentrations of tested samples in the presence of 100 ng/mL LPS. After 24 h incubation, cells were washed twice with DPBS and incubated with 100 μL of 0.25 mg/mL MTT reagent for 3 h. The medium was then removed, and 100 μL dimethyl sulfoxide were added to each well. The resulting color and absorbance were measured at 570 nm to calculate the cell viability.

### 2.14. LPS-Induced NO Inhibition Assay

LPS-induced NO production of RAW264.7 cells was determined by Griess assay [53]. The 100 μL quantity of cells was seeded in 96-well plates at an amount of 5 × 10^4^ cells/well and incubated for 24 h. The cells were then treated with 100 ng/mL LPS with vehicle or different concentrations of the tested sample for 24 h. Then, the supernatant was mixed with the same volume of Griess reagent (2% sulfanilamide in 5% phosphoric acid/0.2% *N*-1-naphthylethylenediamine dihydrochloride in distilled water = 1:1) and incubated at room temperature for 10 min. The absorbance was measured at 540 nm to calculate the NO inhibition. Sodium nitrite was used to generate a standard curve.

### 2.15. Western Blot Analysis

Cells were seeded into a 6-well plate with a cell density of 4 × 10^6^ cells/well and incubated for 24 h. The cells were then treated with 100 ng/mL LPS with vehicle or different concentrations of the tested compound for 24 h. After incubation, the supernatant was removed, and the cells were washed with PBS. Then, the cells were collected and lysed in an ice-cold lysis buffer on ice for 30 min; this was followed by centrifugation at 10,000 rpm for 30 min at 4 °C, and the supernatant was collected as the protein sample solutions for Western blot analysis. The SDS-PAGE was prepared using separating gel (10% polyacrylamide) and stacking gel (5% polyacrylamide). The protein sample solutions were mixed with RIPA buffer and sample dye; this was followed by 100 °C heating for 5 min, before they were cooled on ice. Equal amounts of protein were loaded for electrophoresis. Then, polyvinylidene difluoride (PVDF) was used to transfer proteins. Then, 1 h of blocking of PVDF was performed using blocking buffer (2% BSA in TBST) at room temperature, followed by incubation with primary antibody at 4 °C overnight. The PVDF was washed by TBST; this was followed by 1 h incubation with the secondary antibody at room temperature. Enhanced chemiluminescence (ECL) was used to visualize the protein, and the intensity was captured using a chemiluminescence imaging system.

### 2.16. Molecular Modeling Docking Study

A previously reported method was utilized to perform the molecular docking models [54,55]. The 2D structures of the ligands were constructed using ChemDraw 16.0 (PerkinElmer, Waltham, MA, USA) and converted to 3D structures. Energy minimization of the ligands was conducted using the MM2 force field in Chem3D 16.0. AutoDock Tools 1.5.7 (Scripps Research, La Jolla, CA, USA) was used to prepare the docking input files. The crystal structures of the target proteins were obtained from the RCSB Protein Data Bank. AutoDock Vina 1.1.2 (Scripps Research, La Jolla, CA, USA) was utilized to perform the molecular docking, and the binding mode with the lowest predicted free energy was selected as the most stable conformation. Discovery Studio 2021 (BIOVIA, San Diego, CA, USA) was used to visualize the intermolecular interactions of the most stable complexes.

### 2.17. Statistical Analysis

Statistical significance among groups was assessed using one-way analysis of variance (ANOVA), followed by Tukey’s post hoc test for multiple comparisons. Statistical analysis was performed using IBM SPSS Statistics (version 29.0). Different letters (A, B, C, etc.) indicate statistically significant differences among groups (*p* < 0.05, Tukey’s test). All experiments were conducted in triplicate, and data are expressed as mean ± standard deviation (SD).

## 3. Results

### 3.1. Determination of TPC, TFC, and Yields of Solvent Extracts

The total phenolic content (TPC), total flavonoid content (TFC), and yields of solvent extracts from the leaves of *E. japonica* were shown in Table 1. According to the results, the yields of various solvent extracts ranged from 1.32% to 15.72%, and the methanol (15.72%) exhibited the highest extraction yield, followed by 100 °C water (14.52%) and water (12.16%). The *n*-hexane, ethyl acetate, and acetone extracts exhibited lower extraction yield, aligning with the relative polarity. On the other hand, the TPC results indicated that the water 100 °C extract (37.56 ± 1.54 mg/g) and methanol extract (35.59 ± 2.82 mg/g) possessed the highest total phenolic content, indicating that the extraction of phenolic compounds might align with the higher relative polarity of extract solutions. In contrast, the extract solutions with lower relative polarity showed higher TFC, including ethyl acetate (71.42 ± 9.97 mg/g), acetone (65.01 ± 5.87 mg/g), and *n*-hexane (64.30 ± 8.42 mg/g).

### 3.2. DPPH Scavenging Capability of Solvent Extracts

According to the results, as shown in Table 2, most of the solvent extracts possessed superior DPPH scavenging activity compared to the positive control, BHT (SC_50_ = 230.05 ± 25.40 μg/mL). Among the extracts, the 100 °C water extract exhibited the strongest DPPH scavenging capability, with an SC_50_ value of 98.84 ± 10.42 μg/mL, followed by methanol (114.11 ± 3.68 μg/mL), water (192.74 ± 18.48 μg/mL), and ethanol (222.44 ± 16.87 μg/mL).

### 3.3. ABTS Scavenging Capability of Solvent Extracts

In the results of ABTS scavenging (Table 2), the methanol extract exhibited the most potent ABTS scavenging activity, with an SC_50_ value of 35.42 ± 0.60 μg/mL, followed by the 100 °C water extract (56.22 ± 5.16 μg/mL), water extract (58.80 ± 4.19 μg/mL), ethanol extract (63.59 ± 2.95 μg/mL), and acetone extract (77.20 ± 3.94 μg/mL). Although all solvent extracts displayed lower ABTS scavenging activities (SC_50_ = 35.42 ± 0.60–247.35 ± 12.89 μg/mL) compared to the positive control, BHT (SC_50_ = 29.95 ± 1.04 μg/mL), the methanol extract demonstrated comparable radical scavenging capability, highlighting its antioxidant potential.

### 3.4. Superoxide Scavenging Capability of Solvent Extracts

According to the superoxide scavenging results (Table 2), the 100 °C water extract exhibited the strongest superoxide radical scavenging activity among the solvent extracts, with an SC_50_ value of 98.84 ± 10.42 μg/mL, followed by the water extract (SC_50_ = 63.68 ± 5.17 μg/mL) and methanol extract (SC_50_ = 191.27 ± 24.04 μg/mL). In contrast, *n*-hexane, ethyl acetate, acetone, and ethanol extracts, which have lower relative polarities, did not show superoxide scavenging activity (SC_50_ > 400 μg/mL).

### 3.5. Ferric Reducing Antioxidant Power of Solvent Extracts

The FRAP results are presented in Table 2. All solvent extracts demonstrated potent FRAP activity. Among them, the 100 °C water extract exhibited the highest FRAP value, with a TE of 882.65 ± 32.62 mM/g, followed by methanol (738.49 ± 33.54 mM/g), water (628.09 ± 43.85 mM/g), ethanol (456.85 ± 23.37 mM/g), and ethyl acetate extracts (358.09 ± 14.20 mM/g).

Based on the above antioxidant analysis results, the data indicate that the 100 °C water extract exhibited the strongest antioxidant activity among the solvent extracts across various assays, including DPPH, ABTS, superoxide scavenging, and FRAP. Methanol and water extracts also demonstrated significant antioxidant potential. Furthermore, the antioxidant activity showed a strong correlation with the phenolic contents of the extracts, suggesting that TPC of *E. japonica* plays a crucial role as a major contributor to the observed antioxidant effects.

### 3.6. α-Glucosidase Inhibition of Solvent Extracts

For the α-glucosidase inhibitory analysis (Table 3), all solvent extracts exhibited significantly stronger inhibitory activity compared to the positive control, acarbose (IC_50_ = 197.04 ± 5.70 μg/mL). Among them, the ethyl acetate extract possessed the most potent inhibition, with an IC_50_ value of 23.45 ± 1.84 μg/mL, representing an 8.4-fold stronger activity than acarbose. This was followed by ethanol (28.67 ± 2.24 μg/mL), water (44.18 ± 3.53 μg/mL), and methanol extracts (50.40 ± 2.80 μg/mL).

### 3.7. Acetylcholinesterase (AChE) Inhibition of Solvent Extracts

According to the results in Table 3, most solvent extracts exhibited stronger AChE inhibition than the positive control, chlorogenic acid (IC_50_ = 136.93 ± 11.78 μg/mL). Among the extracts, the *n*-hexane extract showed the most potent AChE inhibitory activity, with an IC_50_ value of 54.37 ± 3.35 μg/mL, followed by ethyl acetate (65.15 ± 5.87 μg/mL), methanol (76.52 ± 1.46 μg/mL), and ethanol extracts (106.53 ± 9.02 μg/mL).

### 3.8. Antioxidant Properties of Isolated Components

To further investigate the bioactive potential of *E. japonica*, six components, including oleanolic acid (**1**), ursolic acid (**2**), corosolic acid (**3**), tormentic acid (**4**), epicatechin (**5**), and rutin (**6**), were isolated from ethyl acetate extract of *E. japonica* and evaluated for their bioactivities. DPPH, ABTS, superoxide scavenging, and FRAP analysis were conducted to evaluate the antioxidant properties (Table 4). The results revealed that triterpenoids, including oleanolic acid (**1**), ursolic acid (**2**), corosolic acid (**3**), and tormentic acid (**4**), showed limited antioxidant activity. Notably, epicatechin (**5**) and rutin (**6**) showed potency in DPPH, ABTS, superoxide scavenging, and FRAP. Particularly, epicatechin (**5**) exhibited significant DPPH and ABTS scavenging activities, with SC_50_ values of 56.94 ± 0.91 and 7.83 ± 0.34 μM, respectively, surpassing the positive control, BHT. On the other hand, rutin (**6**) performed with remarkable superoxide scavenging activities, with a SC_50_ values of 6.69 ± 0.25 μM, significantly stronger than the positive control, cymaroside (SC_50_ = 38.33 ± 0.69). Furthermore, FRAP analysis demonstrated that epicatechin (**5**) and rutin (**6**) possessed ferric reducing activity, with TE values of 1694.43 ± 21.45 and 1609.10 ± 25.67 mM/g, respectively.

### 3.9. α-Glucosidase Inhibition of Isolated Compounds

In the α-glucosidase inhibition results (Table 5), triterpenoids, including oleanolic acid (**1**), ursolic acid (**2**), corosolic acid (**3**), and tormentic acid (**4**), demonstrated more potent α-glucosidase inhibitory activity than epicatechin (**5**) and rutin (**6**). Among the isolated components, ursolic acid (**2**) (IC_50_ = 10.68 ± 0.76 μM) exhibited the most potent inhibition against α-glucosidase, followed by corosolic acid (**3**) (IC_50_ = 13.83 ± 2.11 μM) and oleanolic acid (**1**) (IC_50_ = 21.10 ± 1.48 μM). Notably, ursolic acid (**2**) displayed a 39-fold stronger activity than the positive control, acarbose (IC_50_ = 419.93 ± 29.15 μM).

### 3.10. Acetylcholinesterase (AChE) Inhibition of Isolated Compounds

According to the AChE inhibition results in Table 5, triterpenoids, including oleanolic acid (**1**), ursolic acid (**2**), corosolic acid (**3**), and tormentic acid (**4**), exhibited stronger AChE inhibitory activities compared to the positive control, chlorogenic acid (IC_50_ = 320.25 ± 4.13 μM). Among the isolated components, tormentic acid (**4**) demonstrated the most potent AChE inhibitory activity, with an IC_50_ value of 281.05 ± 8.55 μM, followed by corosolic acid (**3**) (IC_50_ = 282.81 ± 16.97 μM), oleanolic acid (**1**) (IC_50_ = 306.78 ± 12.14 μM), and ursolic acid (**2**) (IC_50_ = 310.82 ± 7.31 μM).

### 3.11. Anti-Inflammatory Activity of Isolated Compounds

To further extend the biological effects of these bioactive components, the isolated components were subjected to an assessment of the inhibition of LPS-induced nitric oxide (NO) production in RAW264.7 cells. The quercetin was used as a comparison. According to the results in Table 6, oleanolic acid (**1**), tormentic acid (**4**), and epicatechin (**5**) showed unfavorable effects on inhibition of NO production, while corosolic acid (**3**) exhibited cytotoxicity at the tested concentration (Figure S7). Notably, ursolic acid (**2**) (IC_50_ = 20.18 ± 1.46 μM) exhibited the best NO inhibition against LPS-induced RAW264.7 cells, followed by rutin (**6**) (IC_50_ = 29.47 ± 1.19 μM). Furthermore, the NO inhibitory activity of ursolic acid (**2**) was also comparable, compared to the positive control, quercetin (IC_50_ = 17.05 ± 1.63 μM).

### 3.12. Western Blot Analysis of iNOS Inhibition

**Figure 3:**
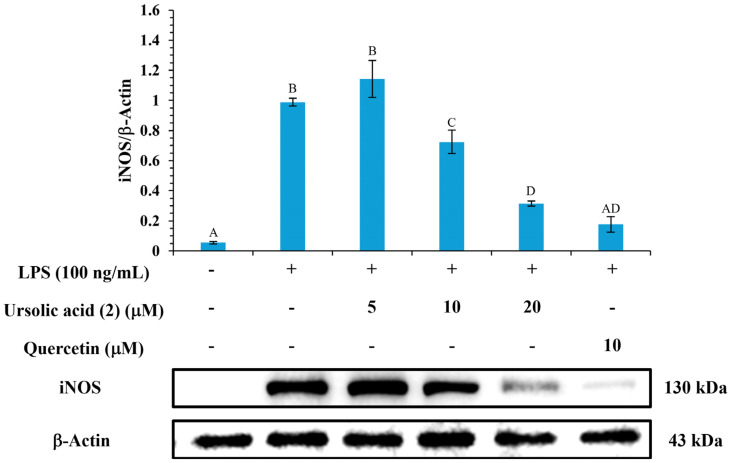
Effects of ursolic acid (**2**) and quercetin on expression of iNOS. Different letters (A, B, C, etc.) indicate statistically significant differences among groups (*p* < 0.05, Tukey’s test).

To further investigate the anti-inflammatory activity of the isolated bioactive component, the most potent component, ursolic acid (**2**), was evaluated for its effect on protein expression levels of iNOS by Western blot analysis in LPS-stimulated RAW264.7 cells. The quercetin was used as a comparison. Based on the results, 100 ng/mL LPS significantly induced the expression of iNOS (LPS group). In contrast, the negative control group incubated without 100 ng/mL LPS showed low expression of iNOS. Notably, ursolic acid (**2**) significantly inhibited 26.7% and 68.1% of iNOS expression at concentrations of 10 and 20 μM, respectively, compared to the LPS group, demonstrating a dose-dependent relationship (Figure 3).

### 3.13. Molecular Docking Analysis

According to the results of enzyme inhibition (Table 5), the potent anti-*glucosidase* agents, including oleanolic acid (**1**), ursolic acid (**2**), and corosolic acid (**3**), were subjected to further molecular docking study. Since the 3D structural information of α-glucosidase from *S. cerevisiae* was unclear, the crystal structure of isomaltase (PDB ID: 3A4A) from *S. cerevisiae*, with an 84% sequence similarity to α-glucosidase, was chosen as the target protein for this docking model. These three compounds were applied over the active site of the target protein (PDB ID: 3A4A) to evaluate the binding affinity study. Autodock vina 1.1.2 (Scripps Research, La Jolla, CA, USA) was utilized to calculate the binding energy between the ligands and the target protein.

According to the binding affinity results (Table 7), ursolic acid (**2**) (−8.7 kcal/mol) exhibited the strongest binding energy to the active binding site of *α*-glucosidase among the bioactive components, followed by corosolic acid (**3**) (−8.6 kcal/mol) and oleanolic acid (**1**) (−8.4 kcal/mol). Furthermore, the potent anti-*α*-glucosidase agents, including oleanolic acid (**1**), ursolic acid (**2**), and corosolic acid (**3**), demonstrated more stable binding energy at the active binding site of *α*-glucosidase compared to the acarbose (−5.1 kcal/mol), consistent with the results of enzyme inhibition.

**Figure 4:**
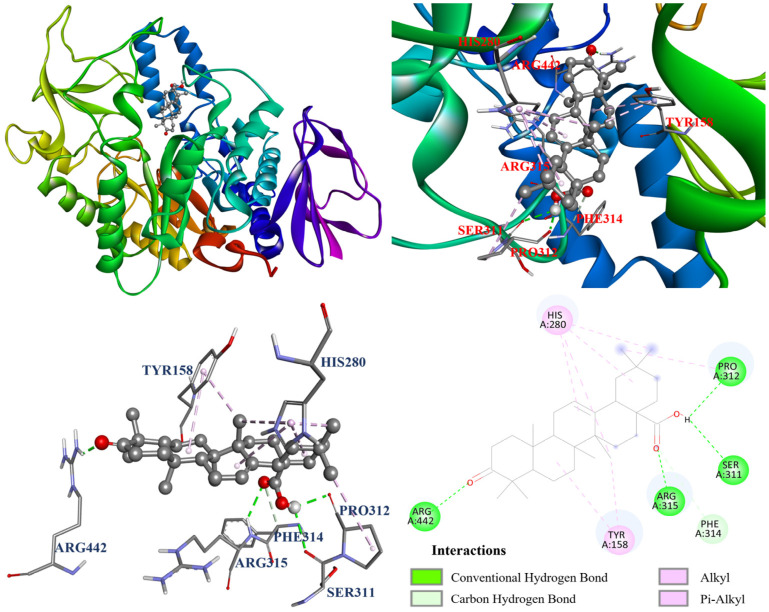
The interaction of oleanolic acid (**1**) with the α-glucosidase active binding sites.

**Figure 5:**
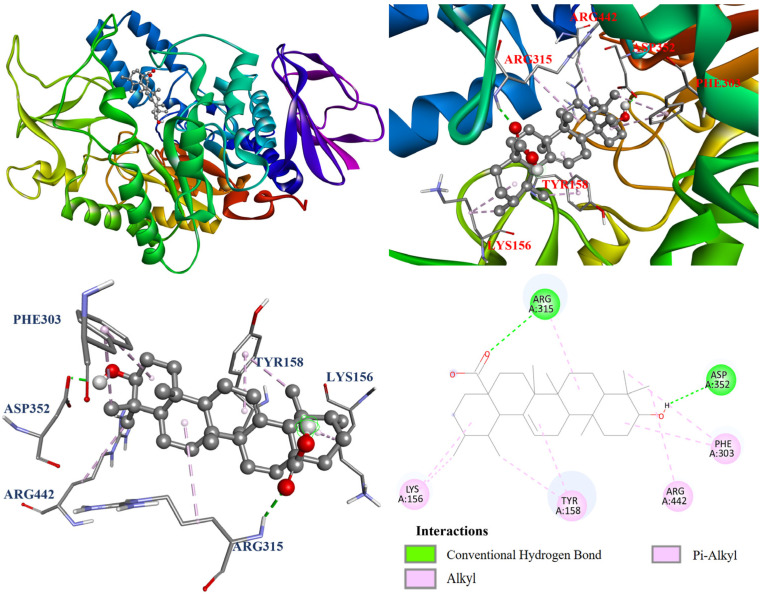
The interaction of ursolic acid (**2**) with the α-glucosidase active binding sites.

**Figure 6:**
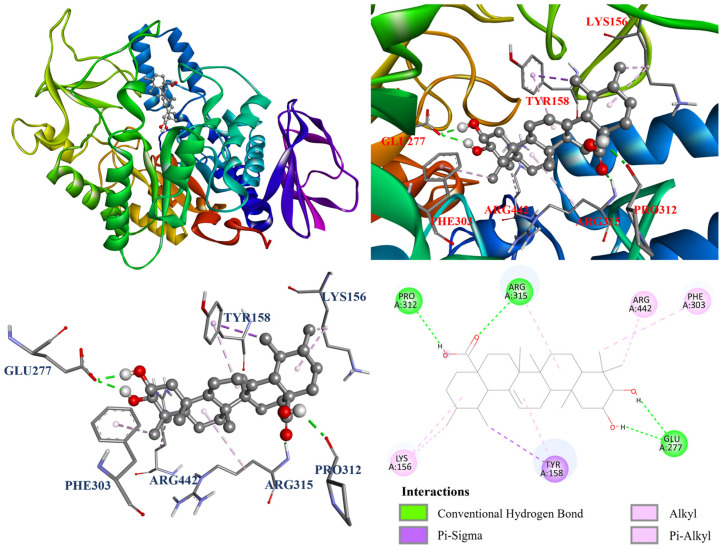
The interaction of corosolic acid (**3**) with the α-glucosidase active binding sites.

The interactions between the protein and ligand in the molecular docking model were further visualized by Discovery studio 2021 (Accelrys Software, Inc.; San Diego, CA, USA). The docking model of oleanolic acid (**1**) with PDB: 3A4A is shown in Figure 4. According to the results, oleanolic acid (**1**) formed four conventional hydrogen bonds with Ser311, Pro312, Arg315, and Arg442; one carbon hydrogen bond with Phe314; and seven alkyl and π–alkyl interactions with Tyr158, His280, and Pro312. On the other hand, Figure 5 demonstrated that ursolic acid (**2**) formed two conventional hydrogen bonds with Arg315 and Asp352; and eight alkyl and π–alkyl interactions with Lys156, Tyr158, Phe303, Arg315, and Arg442. As shown in Figure 6, corosolic acid (**3**) formed four conventional hydrogen bonds with Glu277, Pro312, and Arg315; one π–sigma interaction with Tyr158; and six alkyl and π–alkyl interactions with Lys156, Tyr158, Phe303, Arg315, and Arg442.

To further investigate the binding mechanism between bioactive components and the acetylcholinesterase active binding site, the molecular docking model of the bioactive components, including oleanolic acid (**1**), ursolic acid (**2**), corosolic acid (**3**), and tormentic acid (**4**), was rendered with the AChE crystal structure (PDB ID: 1C2B). As shown in Table 8, tormentic acid (**4**) (−8.8 kcal/mol) displayed the strongest binding affinity, followed by corosolic acid (**3**) (−8.6 kcal/mol), oleanolic acid (**1**) (−8.2 kcal/mol), and ursolic acid (**2**) (−7.8 kcal/mol). Additionally, the more stable binding energies of potent compounds relative to the positive control, chlorogenic acid (−7.8 kcal/mol), were also observed, demonstrating the AChE inhibition results.

**Figure 7:**
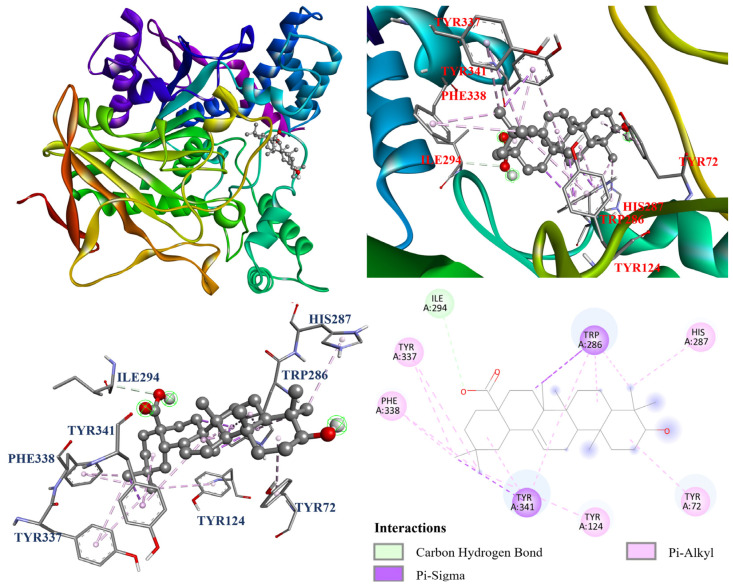
The interaction of oleanolic acid (**1**) with the AChE active binding sites.

**Figure 8:**
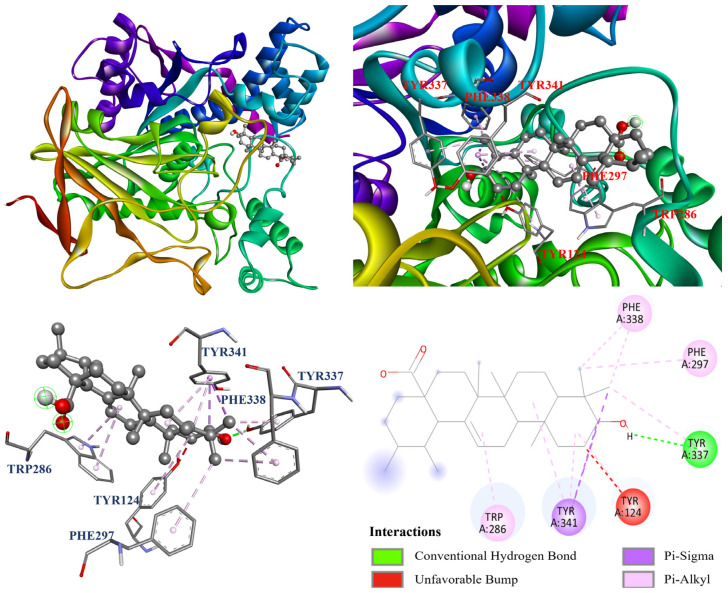
The interaction of ursolic acid (**2**) with the AChE active binding sites.

**Figure 9:**
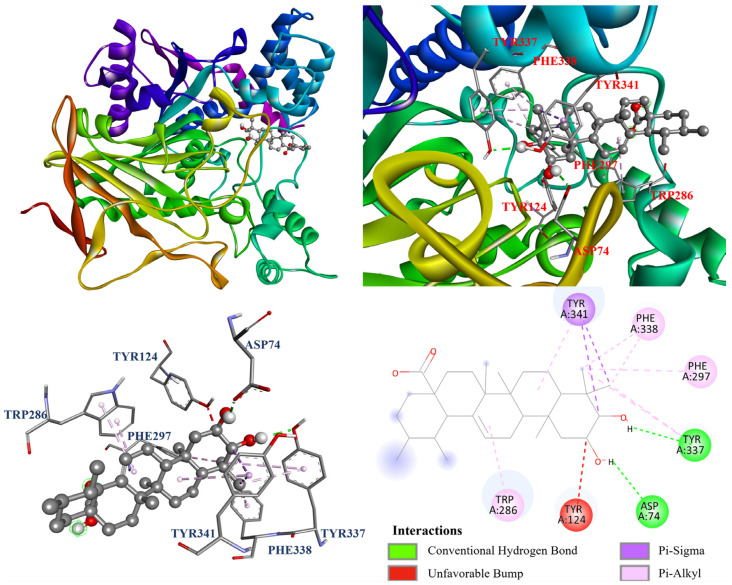
The interaction of corosolic acid (**3**) with the AChE active binding sites.

**Figure 10:**
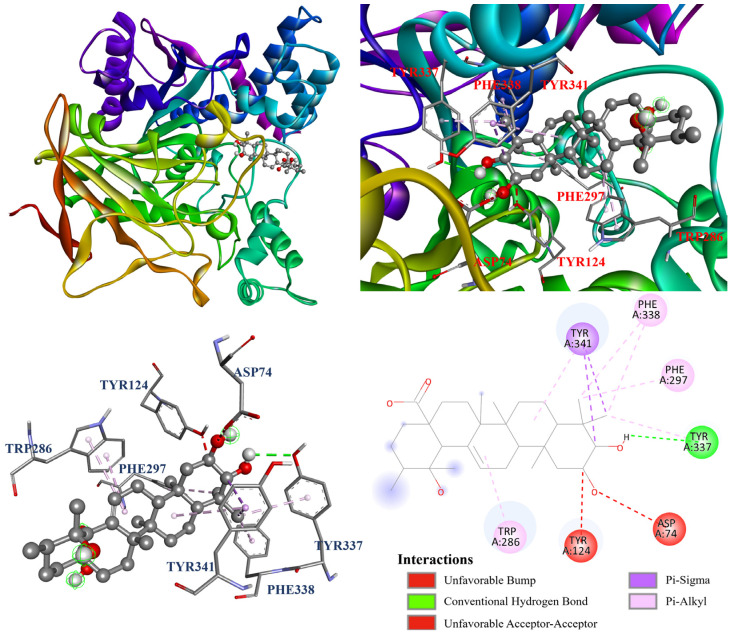
The interaction of tormentic acid (**4**) with the AChE active binding sites.

Furthermore, the molecular docking models of compounds **1**–**4** were visualized and are illustrated in Figure 7, Figure 8, Figure 9 and Figure 10 . As shown in Figure 7, oleanolic acid (**1**) displayed one carbon hydrogen bond with Ile294; two π–σ interactions with Trp286 and Tyr341; and twelve π–alkyl interactions with Tyr72, Tyr124, Trp286, His287, Tyr337, Phe338, and Tyr341. In Figure 8, ursolic acid (**2**) forms one Tyr337; two π–σ interactions with Tyr341; and eight π–alkyl interactions with Tyr124, Trp286, Phe297, Tyr337, Phe338, and Tyr341, while it also forms an unfavorable bump with Tyr124. Additionally, Figure 9 shows that corosolic acid (**3**) formed two conventional hydrogen bonds with Asp74 and Tyr337; two π–σ interactions with Tyr341; and seven π–alkyl interactions with Trp286, Phe297, Tyr337, Phe338, and Tyr341, while it also formed an unfavorable bump with Tyr124. In case of Figure 10, tormentic acid (**4**) forms one conventional hydrogen bond with Tyr337; two π–σ interactions with Tyr341; and six π–alkyl interactions with Trp286, Phe297, Tyr337, Phe338, and Tyr341, while it also forms an unfavorable bump and one unfavorable acceptor–acceptor interaction with Tyr124 and Asp74, respectively.

To further investigate the mechanism of NO inhibition, the molecular docking modeling for ursolic acid (**2**) and rutin (**6**) was conducted with the iNOS crystal structure (PDB ID: 1M9T). As shown in Table 9, ursolic acid (**2**) (−7.5 kcal/mol) and rutin (**6**) (−8.9 kcal/mol) exhibited potent binding affinity with iNOS. Additionally, the similar binding energies of ursolic acid (**2**), compared to the positive control, quercetin (−7.7 kcal/mol), were also observed.

**Figure 11:**
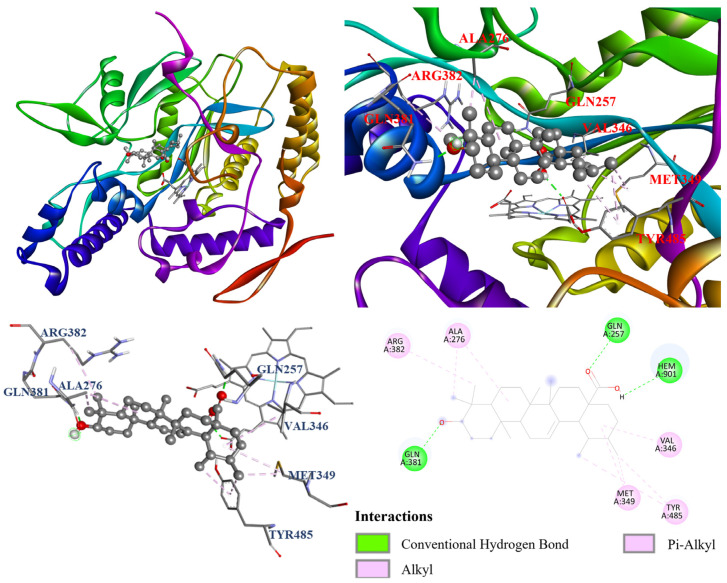
The interaction of ursolic acid (**2**) with iNOS.

**Figure 12:**
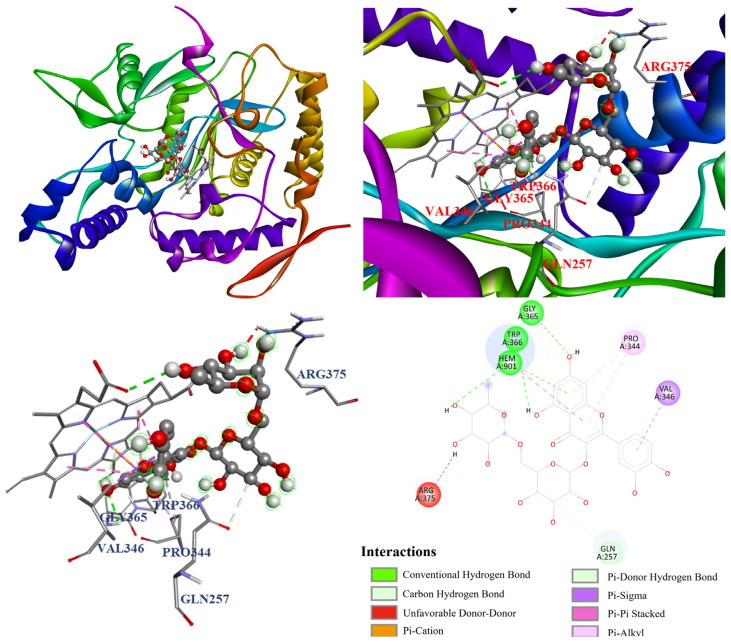
The interaction of rutin (**6**) with iNOS.

Furthermore, the molecular docking models of ursolic acid (**2**) and rutin (**6**) were also visualized and are illustrated in Figure 11 and Figure 12. As shown in Figure 10, ursolic acid (**2**) displayed three conventional hydrogen bonds with Gln257, Gln381, and Hem901; and eight π–alkyl and alkyl interactions with Ala276, Val346, Met349, Arg382, and Tyr485. Additionally, Figure 11 shows that rutin (**6**) performed three conventional hydrogen bonds with Gly365, Trp366, and Hem901; a π–donor hydrogen bond and carbon hydrogen bond with Gln257; π–σ interaction with Val346; a π–π stacked interaction with Hem901; π–cation interaction with Hem901; and two π–alkyl interactions with Pro344. Notably, there is an unfavorable donor–donor interaction with Arg375, which resulted in its lower NO inhibition relative to ursolic acid (**2**).

## 4. Discussions

The selection of extraction solvents is a critical factor in natural product research, as it significantly influences the yield and bioactivity of bioactive components by aligning with the properties and polarities of the target compounds [56]. In this study, it was demonstrated that leaves of *Eriobotrya japonica* possessed significant bioactive potential, with solvent extracts and isolated compounds showing varied effects in antioxidant and enzyme inhibition assays. The radical (including DPPH, ABTS, and superoxide) scavenging and FRAP results indicated that the phenolic content encompassed major potential antioxidants in leaves of *E. japonica*, particularly. The methanol and water 100 °C extracts and epicatechin (**5**) exhibited significant antioxidant activities, demonstrating that phenolic compounds contribute substantially to antioxidant capacity with their redox properties, acting as reducing agents, hydrogen donors, and single oxygen quenchers. The high levels of antioxidant activities and TPC in the methanol and water 100 °C extracts suggest that polar protic solvents efficiently extract phenolic-rich components in *E. japonica*.

In T2DM, α-glucosidase is considered to be a therapeutic target for regulating the level of hyperglycemia [57,58]. In the results from the α-glucosidase inhibition, ethyl acetate and ethanol extracts demonstrated superior activities relative to acarbose; the latter is commonly used in managing postprandial hyperglycemia in diabetic patients. The notable inhibitions of α-glucosidase by these extracts suggest that leaves of *E. japonica* may offer a natural source of α-glucosidase inhibitors. Among the isolated compounds, triterpenoids, including oleanolic acid (**1**), ursolic acid (**2**), and corosolic acid (**3**), were more potent than epicatechin (**5**), rutin (**6**), and acarbose, with significantly lower IC_50_ values. Molecular docking studies further supported these findings, showing strong binding affinities and interactions with the α-glucosidase active site, reinforcing the potent applications of these compounds as natural alternatives to anti-α-glucosidase agents.

Neurodegenerative disorder is the most common cause of dementia, which leads to the deterioration of critical cognitive functions such as memory, comprehension, and speech [59,60]. Acetylcholine plays a crucial role in cognitive processes such as learning and memory, which becomes an important therapeutic target for treating neurodegenerative disorders [61,62]. On the other hand, chlorogenic acid, a phenolic acid derived from a natural source, has been shown to have neuroprotective properties and an AChE inhibitory effect associated with Alzheimer’s disease [63]. Therefore, chlorogenic acid was used as positive control. In the acetylcholinesterase (AChE) inhibition results, it was demonstrated that *n*-hexane and ethyl acetate extracts from *E. japonica* effectively inhibit AChE, with tormentic acid (**4**) demonstrating particularly notable inhibitory activity. Molecular docking revealed that tormentic acid (**4**) interacts strongly with critical residues in the AChE active site, suggesting a mechanism similar to those of the reported anti-AChE agents used in Alzheimer’s disease management. This inhibition, coupled with the compound’s natural antioxidant properties, indicated a dual potential for neuroprotection. The lipophilic nature of tormentic acid (**4**) and related triterpenes could also facilitate blood–brain barrier penetration [64], an essential factor for neuroactive agents considered as natural alternatives or complementary treatments for neurodegenerative conditions.

The inflammatory response is an essential biological mechanism for protecting the body against injury and infection [65]. However, excessive or dysregulated inflammation contributes to various diseases [66]. Nitric oxide (NO) is a key mediator of inflammation and plays a dual role in immune defense and tissue damage when overproduced [67]. In the NO inhibition assay, ursolic acid (**2**) demonstrated inhibition comparable to the positive control, quercetin, indicating its significant anti-inflammatory potential. Similarly, rutin (**6**) exhibited notable NO inhibition, though slightly weaker than quercetin. Furthermore, the effect of ursolic acid (**2**) on the reduction of iNOS expression also demonstrated its in vitro anti-inflammatory activity. These results suggest that ursolic acid (**2**) and rutin (**6**) may contribute to the anti-inflammatory properties in leaves of *E. japonica*. Molecular docking further confirmed the strong interactions of ursolic acid (**2**) and rutin (**6**) with key residues of inducible nitric oxide synthase (iNOS), providing insights into their NO inhibition mechanism. Though rutin (**6**) exhibited a more stable affinity than ursolic acid (**2**), the unfavorable donor–donor interaction with Arg375 between rutin (**6**) and iNOS might result in the inferior anti-inflammatory effects of rutin (**6**), compared to ursolic acid (**2**) and quercetin. Based on the observed bioactivities, these findings highlight the potential of *E. japonica* bioactive compounds as natural agents for managing inflammation and oxidative stress, as well as other associated pathways.

In summary, this study highlights the efficacy of different solvents in extracting bioactive compounds from leaves of *E. japonica*, with varying antioxidant, anti-α-glucosidase, and anti-acetylcholinesterase activities, depending on solvent polarity. The methanol, water, and 100 °C water extracts were rich in phenolic compounds, which contributed substantially to their antioxidant capacity. Additionally, the triterpenes isolated from ethyl acetate and ethanol extracts, especially oleanolic acid (**1**), ursolic acid (**2**), and tormentic acid (**3**), showed potential as natural enzyme inhibitors against α-glucosidase and AChE. Moreover, ursolic acid (**2**) was found to possess the most potent anti-inflammatory activity. These findings suggest that leaves of *E. japonica* contain diverse bioactive components with applications in managing oxidative stress, hyperglycemia, and neurodegenerative and inflammatory conditions, providing a foundation for further in vivo studies and development of potent therapeutic agents.

## 5. Conclusions

This study demonstrates that the bioactive extracts and isolated components from leaves of *Eriobotrya japonica* exhibit significant antioxidant properties and anti-inflammatory effects, as well as potent enzyme inhibitory activities against α-glucosidase and AChE, through in vitro and in silico molecular docking analyses. Among the bioactive extracts, the 100 °C water extract showed the highest antioxidant activity, particularly in the DPPH, superoxide, and FRAP assays, followed by the methanol and water extracts. The antioxidant potential was strongly correlated with phenolic content, suggesting that phenolic compounds are major contributors to antioxidant activities.

The α-glucosidase inhibition results demonstrated that ethyl acetate and ethanol extracts exhibited the most potent inhibitory effects, with oleanolic acid (**1**), ursolic acid (**2**), and corosolic acid (**3**) showing IC_50_ values of 21.10 ± 1.48, 10.68 ± 0.76, and 13.83 ± 2.11 μM, respectively, significantly stronger than the acarbose (IC_50_ = 419.93 ± 29.15 μM). The AChE inhibition results revealed that *n*-hexane and ethyl acetate extracts possessed strong inhibitory effects, while triterpenoid compounds **1**–**4** showed AChE inhibition levels comparable to chlorogenic acid. Anti-inflammatory analysis results indicated ursolic acid (**2**) and rutin (**6**) possessed potent anti-inflammatory effects, with IC_50_ values of 20.18 ± 1.46 and 29.47 ± 1.19, respectively, against LPS-induced NO production of RAW264.7 cells. Further, Western blot analysis results indicated that ursolic acid (**2**) expressed significant inhibition against iNOS expression, demonstrating the anti-inflammatory mechanism. Molecular docking further supported these findings, with ursolic acid (**2**) (−8.7 kcal/mol) exhibiting the most stable binding energies, with results superior to acarbose (−5.1 kcal/mol). Triterpenoids, including oleanolic acid (**1**), ursolic acid (**2**), corosolic acid (**3**), and tormentic acid (**4**), showed stronger binding energies compared to chlorogenic acid. Ursolic acid (**2**) and rutin (**6**) exhibited similar binding energies compared to quercetin.

Overall, these findings highlight the biological potential of bioactive components and extracts from leaves of *E. japonica* as a natural source of supplements or candidates for antioxidant, anti-α-glucosidase, anti-AChE, and anti-inflammatory agents, offering promising applications in managing oxidative stress-related diseases, diabetes mellitus, Alzheimer’s disease, and inflammatory disorders.

## Figures and Tables

**Table 1:** TPC, TFC, and extraction yields of different solvents from *E. japonica*.

Extracting Solvents	TPC (mg/g) ^a^ (GAE)	TFC (mg/g) ^b^ (QE)	Yields (%) ^c^
*n*-Hexane	8.11 ± 1.92 ^A^	64.30 ± 8.42 ^C^	1.32
Ethyl acetate	8.50 ± 1.65 ^A^	71.42 ± 9.97 ^C^	4.28
Acetone	15.06 ± 1.35 ^AB^	65.01 ± 5.87 ^C^	5.00
Ethanol	22.00 ± 1.87 ^BC^	44.59 ± 7.31 ^BC^	9.96
Methanol	35.59 ± 2.82 ^DE^	32.06 ± 1.03 ^AB^	15.72
Water	26.70 ± 2.40 ^CD^	13.53 ± 0.91 ^A^	12.16
100 °C Water	37.56 ± 1.54 ^E^	14.57 ± 1.50 ^A^	14.52

^a^ TPC is expressed as mg of gallic acid equivalents (GAE) per g (mg/g). ^b^ TFC is expressed as mg of quercetin equivalents (QE) per gram (mg/g). ^c^ Yield was calculated as (weight of extract/initial weight of dry sample) × 100. Different letters (A, B, C, etc.) indicate statistically significant differences among groups (*p* < 0.05, Tukey’s test). All values are expressed as means ± SD (n = 3).

**Table 2:** The antioxidant activities of different solvent extracts from *E. japonica*.

Extracting Solvents	SC_50_ (μg/mL) ^a^			TE (mM/g) ^b^
	DPPH	ABTS	Superoxide	FRAP
*n*-Hexane	611.84 ± 49.30 ^C^	247.35 ± 12.89 ^E^	>400	156.23 ± 2.31 ^A^
Ethyl acetate	582.60 ± 32.11 ^C^	162.72 ± 8.71 ^D^	>400	358.09 ± 14.20 ^AB^
Acetone	316.92 ± 31.31 ^B^	77.20 ± 3.94 ^C^	>400	151.23 ± 10.15 ^A^
Ethanol	222.44 ± 16.87 ^AB^	63.59 ± 2.95 ^BC^	>400	456.85 ± 23.37 ^ABC^
Methanol	114.11 ± 3.68 ^A^	35.42 ± 0.60 ^AB^	191.27 ± 24.04 ^B^	738.49 ± 33.54 ^BC^
Water	192.74 ± 18.48 ^AB^	58.80 ± 4.19 ^ABC^	63.68 ± 5.17 ^A^	628.09 ± 43.85 ^BC^
100 °C Water	98.84 ± 10.42 ^A^	56.22 ± 5.16 ^ABC^	47.50 ± 5.60 ^A^	882.65 ± 32.62 ^C^
BHT ^c^	230.05 ± 25.40 ^AB^	29.95± 1.04 ^A^	—	3370.27 ± 256.53 ^D^
Cynaroside ^c^	—	—	13.41 ± 2.56 ^A^	—

^a^ The SC_50_ indicates the concentration causing 50% free radical scavenging; ^b^ FRAP is expressed as millimolar (mM) of Trolox equivalents (TE) per gram of extract; ^c^ BHT and cynaroside were applied as positive controls; All data are displayed as mean ± SD (n = 3); Different letters (A, B, C, etc.) indicate statistically significant differences among groups (*p* < 0.05, Tukey’s test).

**Table 3:** Effects of solvent extracts on α-Glucosidase and AChE inhibitions.

Extracting Solvents	IC_50_ (μg/mL) ^a^	
	*α*-Glucosidase	AChE
*n*-Hexane	>400	54.37 ± 3.35 ^A^
Ethyl acetate	23.45 ± 1.84 ^A^	65.15 ± 5.87 ^A^
Acetone	95.61 ± 0.88 ^D^	114.14 ± 10.37 ^BC^
Ethanol	28.67 ± 2.24 ^AB^	106.53 ± 9.02 ^BC^
Methanol	50.40 ± 2.80 ^C^	76.52 ± 1.46 ^AB^
Water	44.18 ± 3.53 ^BC^	138.41 ± 5.75 ^C^
Water 100 °C	96.14 ± 7.19 ^D^	129.36 ± 10.99 ^C^
Acarbose ^b^	197.04 ± 5.70 ^E^	-
Chlorogenic acid ^b^	-	136.93 ± 11.78 ^C^

^a^ The IC_50_ value indicates the concentration causing 50% inhibition; ^b^ Acarbose and chlorogenic acid were used as positive controls; Different letters (A, B, C, etc.) indicate statistically significant differences among groups (*p* < 0.05, Tukey’s test); All data are expressed as mean ± SD (n = 3).

**Table 4:** The antioxidant properties of isolated components from *E. japonica*.

Compounds	SC_50_ (μM) ^a^			TE (mM/g) ^b^
	DPPH	ABTS	Superoxide	FRAP
Oleanolic acid (**1**)	>400	>400	>400	<1
Ursolic acid (**2**)	>400	>400	>400	<1
Corosolic acid (**3**)	>400	>400	>400	<1
Tormentic acid (**4**)	>400	>400	>400	<1
Epicatechin (**5**)	56.94 ± 0.91 ^A^	7.83 ± 0.34 ^A^	210.27 ± 9.46 ^C^	1694.43 ± 21.45 ^A^
Rutin (**6**)	66.30 ± 1.68 ^A^	20.02 ± 0.30 ^B^	6.69 ± 0.25 ^A^	1609.10 ± 25.67 ^A^
BHT ^c^	881.06 ± 70.37 ^B^	25.87 ± 0.01 ^C^	-	3082.65 ± 213.69 ^B^
Cynaroside ^c^	-	-	38.33 ± 0.69 ^B^	-

^a^ The SC_50_ indicates the concentration causing 50% free radical scavenging; ^b^ FRAP is expressed as millimolar (mM) of Trolox equivalents (TE) per g of extract; ^c^ BHT and cynaroside were applied as positive controls; All data are displayed as mean ± SD (n = 3); Different letters (A, B, C) indicate statistically significant differences among groups (*p* < 0.05, Tukey’s test).

**Table 5:** Effects of isolated components from *E. japonica* on α-Glucosidase and AChE inhibitions.

Compounds	IC_50_ (μM) ^a^	
	*α*-Glucosidase	AChE
Oleanolic acid (**1**)	21.10 ± 1.48 ^A^	306.78 ± 12.14 ^A^
Ursolic acid (**2**)	10.68 ± 0.76 ^A^	310.82 ± 7.31 ^A^
Corosolic acid (**3**)	13.83 ± 2.11 ^A^	282.81 ± 16.97 ^A^
Tormentic acid (**4**)	319.89 ± 26.07 ^B^	281.05 ± 8.55 ^A^
Epicatechin (**5**)	>800	404.73 ± 26.34 ^B^
Rutin (**6**)	591.99 ± 10.38 ^D^	451.76 ± 12.88 ^B^
Acarbose ^b^	419.93 ± 29.15 ^C^	-
Chlorogenic acid ^b^	-	320.25 ± 4.13 ^A^

^a^ The IC_50_ value indicates the concentration causing 50% inhibition; ^b^ Acarbose and chlorogenic acid were used as positive controls; Different letters (A, B, C, etc.) indicate statistically significant differences among groups (*p* < 0.05, Tukey’s test); All data are expressed as mean ± SD (n = 3).

**Table 6:** Effects of isolated components on NO production of LPS-induced RAW264.7 cells.

Compounds	NO inhibition IC_50_ (μM) ^a^
Oleanolic acid (**1**)	>50
Ursolic acid (**2**)	20.18 ± 1.46 ^A^
Corosolic acid (**3**)	- ^c^
Tormentic acid (**4**)	>50
Epicatechin (**5**)	>50
Rutin (**6**)	29.47 ± 1.19 ^A^
Quercetin ^b^	17.05 ± 1.63 ^B^

^a^ The IC_50_ value indicates the concentration causing 50% inhibition; ^b^ Quercetin was used as a positive control; ^c^ Cytotoxicity was observed at the tested concentration; Different letters (A and B) indicate statistically significant differences among groups (*p* < 0.05, Tukey’s test); All data are expressed as mean ± SD (n = 3).

**Table 7:** Binding energies of active components with α-glucosidase.

Compounds	Affinity (kcal/mol)
Oleanolic acid (**1**)	−8.4
Ursolic acid (**2**)	−8.7
Corosolic acid (**3**)	−8.6
Acarbose ^a^	−5.1

^a^ Acarbose was used as a positive control.

**Table 8:** Binding energies of active components with AChE.

Compounds	Affinity (kcal/mol)
Oleanolic acid (**1**)	−8.2
Ursolic acid (**2**)	−7.8
Corosolic acid (**3**)	−8.6
Tormentic acid (**4**)	−8.8
Chlorogenic acid ^a^	−7.8

^a^ Chlorogenic acid was used as a positive control.

**Table 9:** Binding energies of active components with iNOS.

Compounds	Affinity (kcal/mol)
Ursolic acid (**2**)	−7.5
Rutin (**6**)	−8.9
Quercetin ^a^	−7.7

^a^ Quercetin was used as a positive control.

## Data Availability

Data are contained within the article and supplementary material.

## Supplementary Materials

The following supporting information can be downloaded at: https://www.mdpi.com/article/10.3390/antiox14040413/s1. Figure S1: The ^1^H-NMR spectrum of oleanolic acid (**1**). Figure S2: The ESI-MS spectrum of oleanolic acid (**1**). Figure S3: The ^1^H-NMR spectrum of ursolic acid (**2**). Figure S4: The ESI-MS spectrum of ursolic acid (**2**). Figure S5: The ^1^H-NMR spectrum of corosolic acid (**3**). Figure S6: The ESI-MS spectrum of corosolic acid (**3**). Figure S7: The ^1^H-NMR spectrum of tormentic acid (**4**). Figure S8: The ESI-MS spectrum of tormentic acid (**4**). Figure S9: The ^1^H-NMR spectrum of epicatechin (**5**). Figure S10: The ESI-MS spectrum of epicatechin (**5**). Figure S11: The ^1^H-NMR spectrum of rutin (**6**). Figure S12: The ESI-MS spectrum of rutin (**6**). Figure S13: Cell viability evaluation of isolated components on RAW264.7 cells.
