# Supplementary material for: Pharmacological and Molecular Docking Investigation of Leaves of Eriobotrya japonica: Antioxidant, Enzyme Inhibition, and Anti-Inflammatory Effects
Source: Antioxidants (Basel). 2025 Mar 29;14(4):413. doi: 10.3390/antiox14040413 (PMC12024191; doi:10.3390/antiox14040413)
Supplement: Supplementary file 1 [file antioxidants-14-00413-s001.zip › antioxidants-3514782-supplementary.pdf]

Supplementary data

Pharmacological and Molecular Docking Investigation of Leaves of *Eriobotrya japonica*: Antioxidant, Enzyme Inhibition, and Anti-inflammatory Effects

Pao-Jen Kuo [1,2], Li-Ting Chen [3,†], Sin-Min Li [3,†], Zih-Rong Chen [4] and Jih-Jung Chen [3,4,5,*]

[1] Department of Plastic Surgery, Kaohsiung Chang Gung Memorial Hospital, Kaohsiung, 833401, Taiwan; bow110470@gmail.com

[2] College of Medicine, Chang Gung University, Taoyuan 333323, Taiwan;

[3] Department of Pharmacy, School of Pharmaceutical Sciences, National Yang Ming Chiao Tung University, Taipei 112304, Taiwan; linting0121@gmail.com (L.-T.C.); samuel147samuel147@gmail.com (S.-M.L.); jjasmine81023@gmail.com (Z.-R.C.)

[4] Department of Medical Research, China Medical University Hospital, China Medical University, Taichung 404333, Taiwan

[5] Traditional Herbal Medicine Research Center, Taipei Medical University Hospital, Taipei 110301, Taiwan

---

* Correspondence: jjungchen@nycu.edu.tw; Tel.: +886-2-2826-7195; Fax: +886-2-2823-2940

† These authors contributed equally to this work.

Contents

Supplementary Fig. 1. The ^{1}H-NMR spectrum (400 MHz, $CDCl_3$) of oleanolic acid (**1**) S3

Supplementary Fig. 2. The ESI-MS spectrum of oleanolic acid (**1**) S3

Supplementary Fig. 3. The ^{1}H-NMR spectrum (400 MHz, $CDCl_3$) of ursolic acid (**2**) S4

Supplementary Fig. 4. The ESI-MS spectrum of ursolic acid (**2**) S4

Supplementary Fig. 5. The ^{1}H-NMR spectrum (400 MHz, methanol-d_4) of corosolic acid (**3**) S5

Supplementary Fig. 6. The ESI-MS spectrum of corosolic acid (**3**) S5

Supplementary Fig. 7. The ^{1}H-NMR spectrum (400 MHz, methanol-d_4) of tormentic acid (**4**) … S6

Supplementary Fig. 8. The ESI-MS spectrum of tormentic acid (**4**) S6

Supplementary Fig. 9. The ^{1}H-NMR spectrum (400 MHz, methanol-d_4) of epicatechin (**5**) S7

Supplementary Fig. 10. The ESI-MS spectrum of epicatechin (**5**) S7

Supplementary Fig. 11. The ^{1}H-NMR spectrum (400 MHz, methanol-d_4) of rutin (**6**) S8

Supplementary Fig. 12. The ESI-MS spectrum of rutin (**6**) S8

Supplementary Fig. 13. Cell viability evaluation of isolated components on RAW264.7 cells……S9

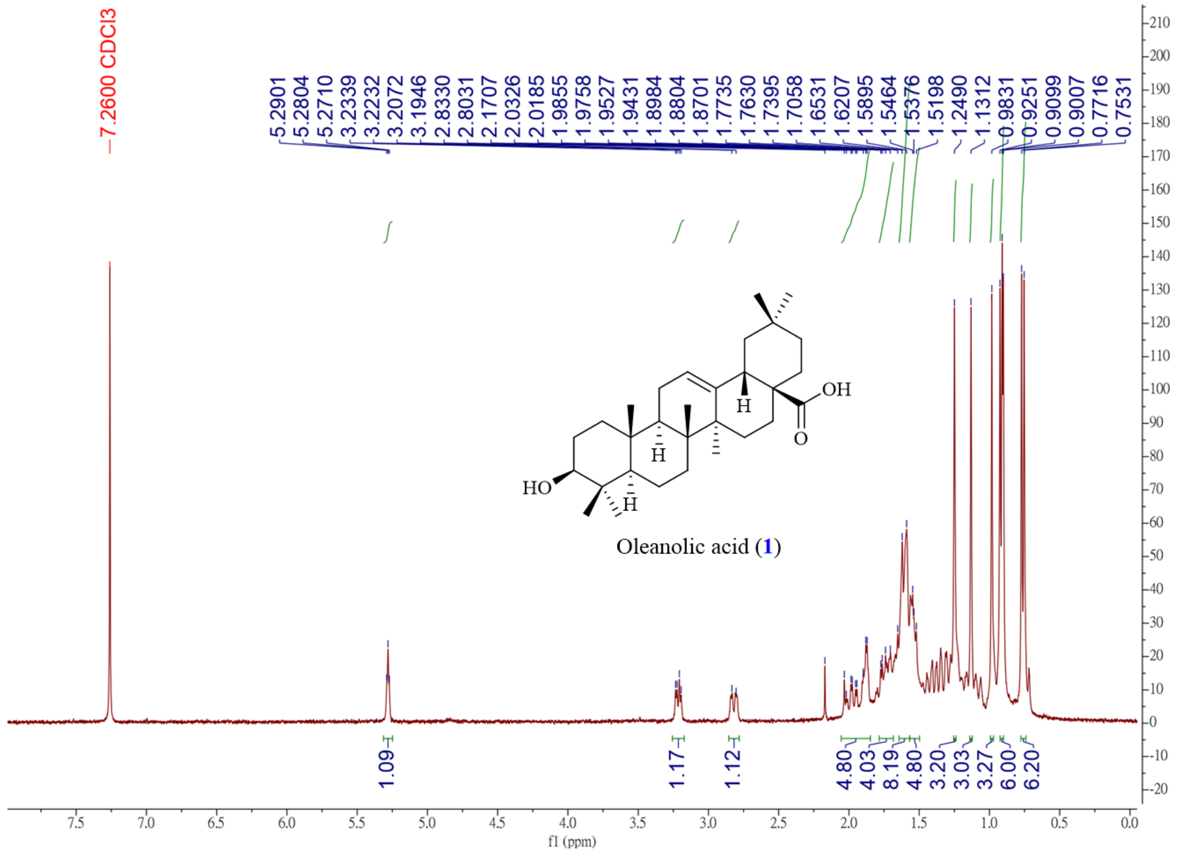

Supplementary Fig. 1. The ^{1}H-NMR spectrum (400 MHz, $CDCl_3$) of oleanolic acid (**1**).

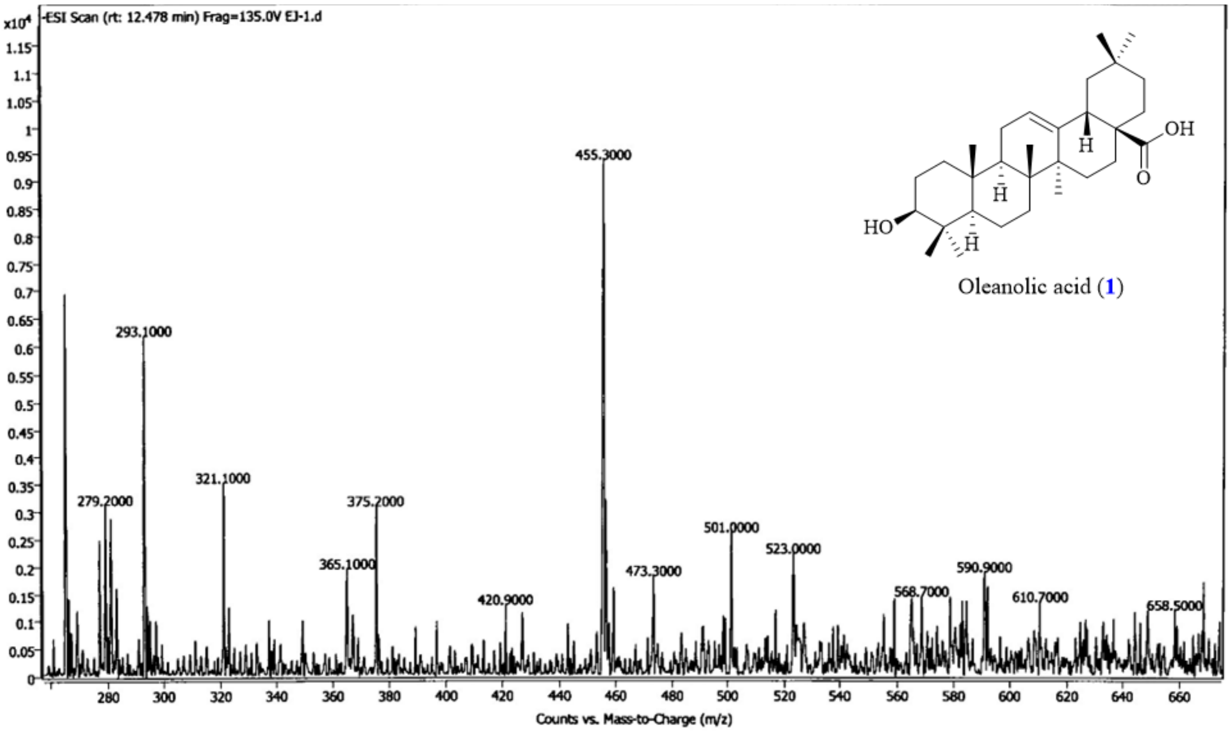

Supplementary Fig. 2. The ESI-MS spectrum of oleanolic acid (**1**).

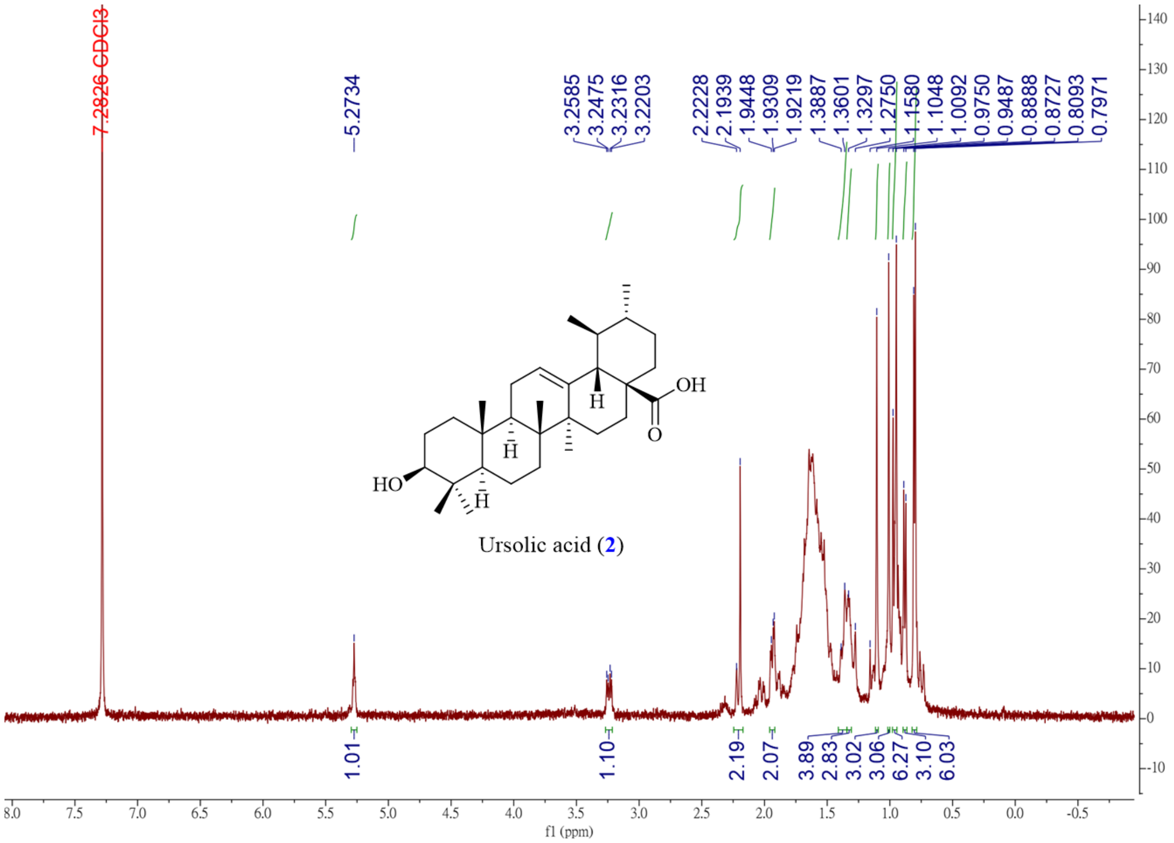

Supplementary Fig. 3. The ¹H-NMR spectrum (400 MHz, $CDCl_3$) of ursolic acid (**2**).

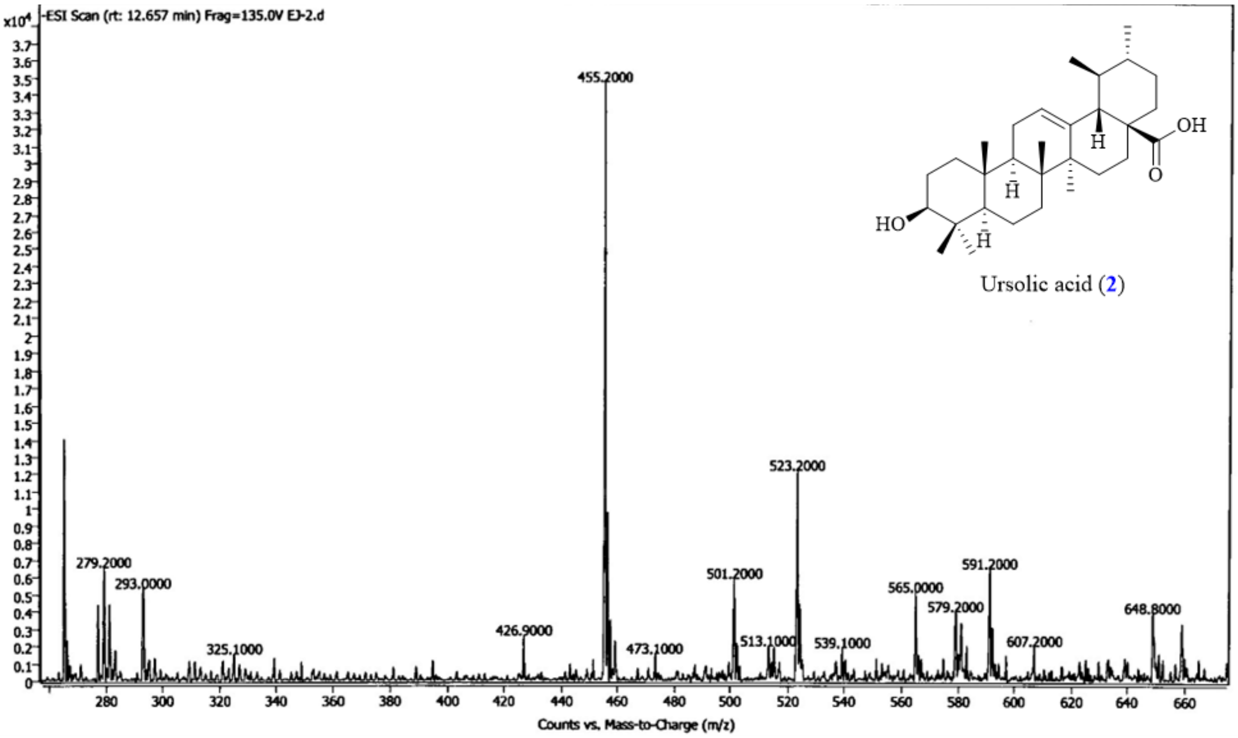

Supplementary Fig. 4. The ESI-MS spectrum of ursolic acid (**2**).

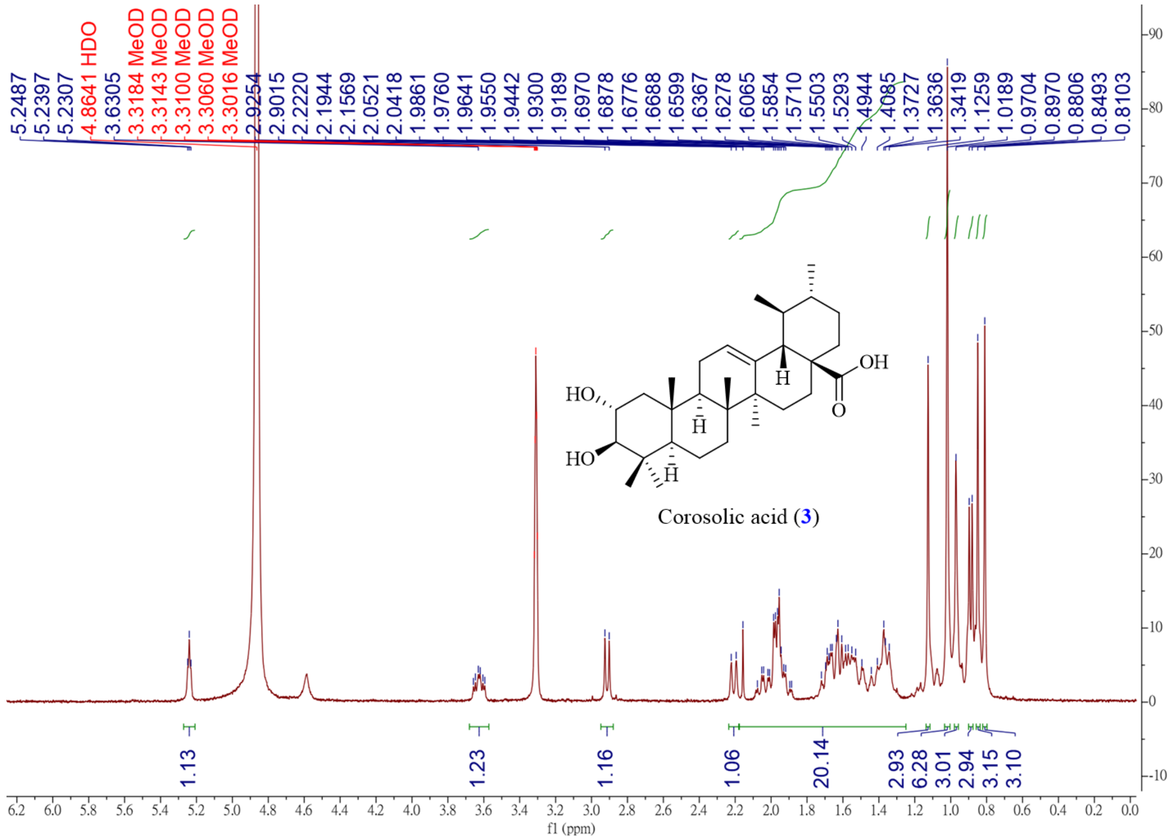

Supplementary Fig. 5. The ^{1}H-NMR spectrum (400 MHz, methanol-d_4) of corosolic acid (**3**).

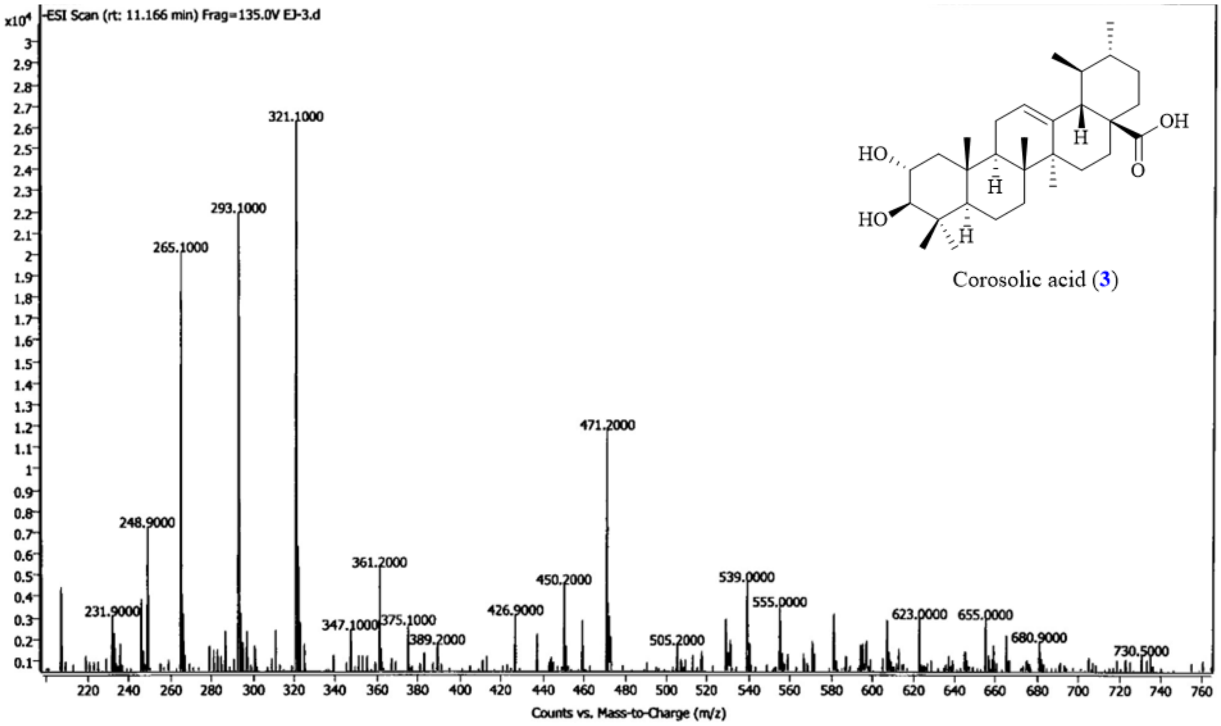

Supplementary Fig. 6. The ESI-MS spectrum of corosolic acid (**3**).

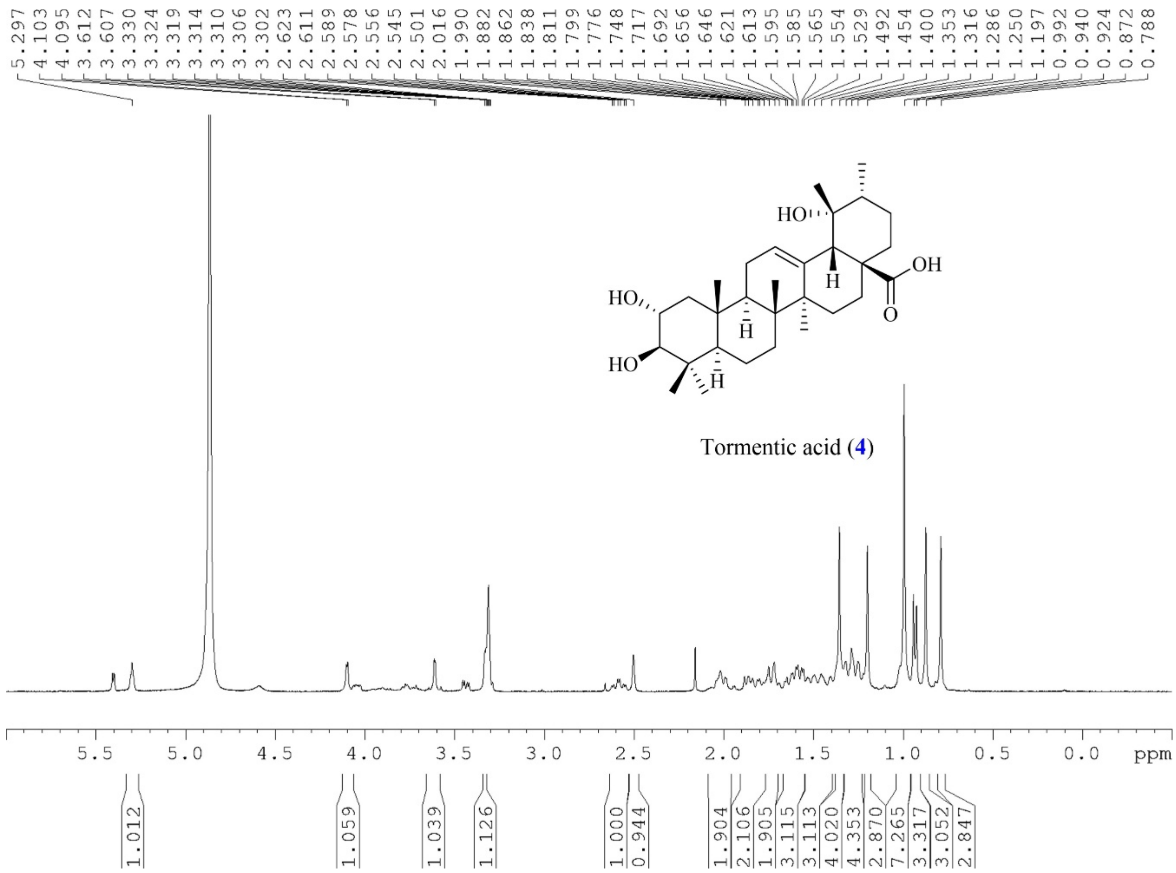

Supplementary Fig. 7. The ^{1}H-NMR spectrum (400 MHz, methanol-d_4) of tormentic acid (**4**).

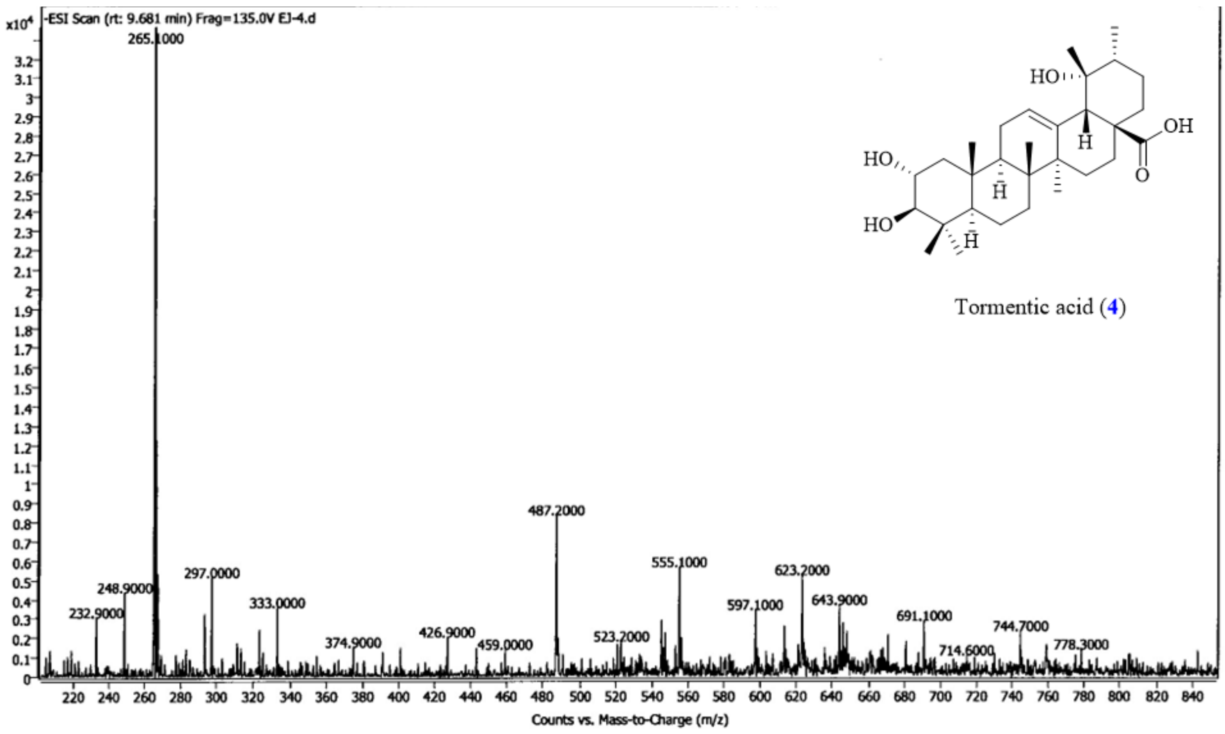

Supplementary Fig. 8. The ESI-MS spectrum of tormentic acid (**4**).

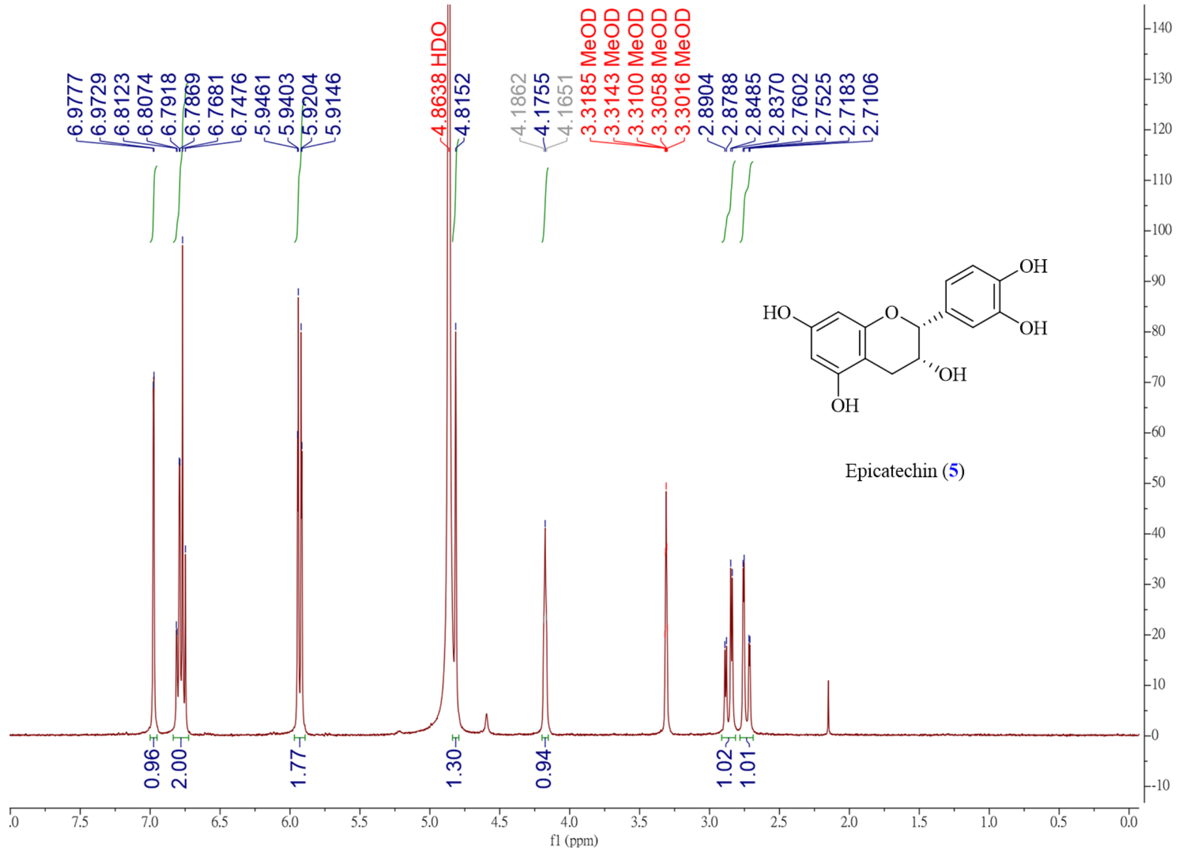

Supplementary Fig. 9. The ^{1}H-NMR spectrum (400 MHz, methanol-d_4) of epicatechin (**5**).

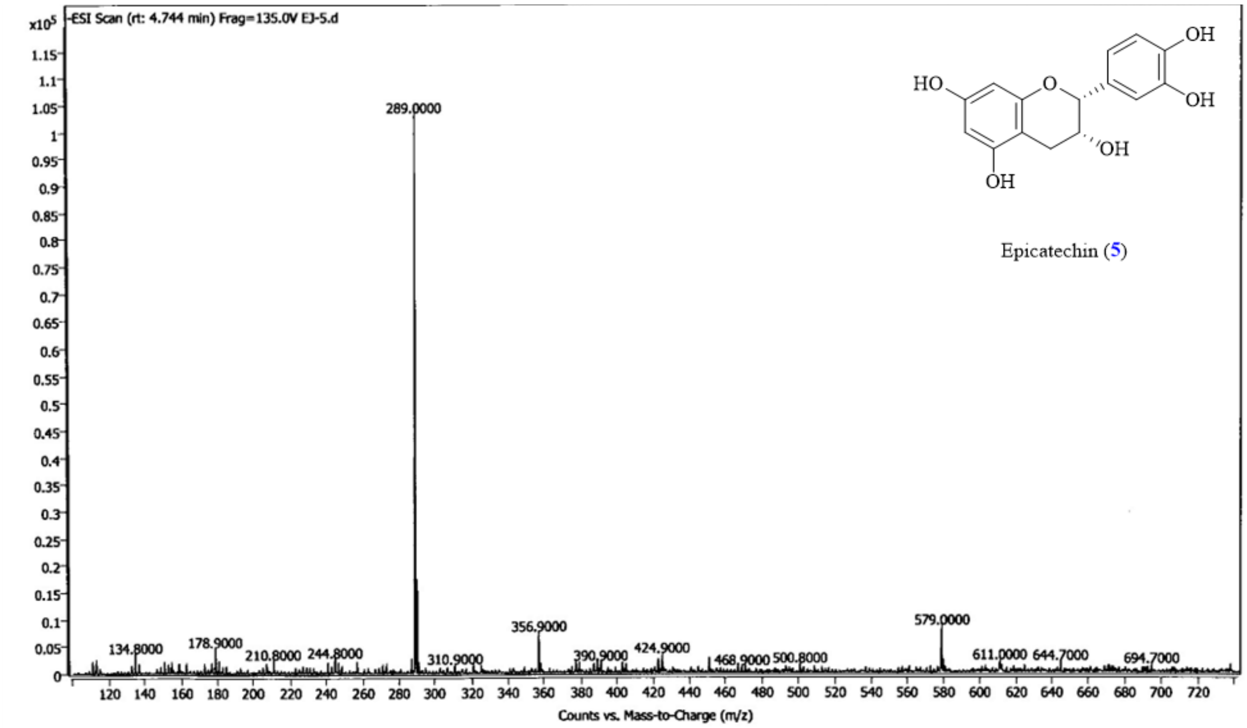

Supplementary Fig. 10. The ESI-MS spectrum of epicatechin (**5**).

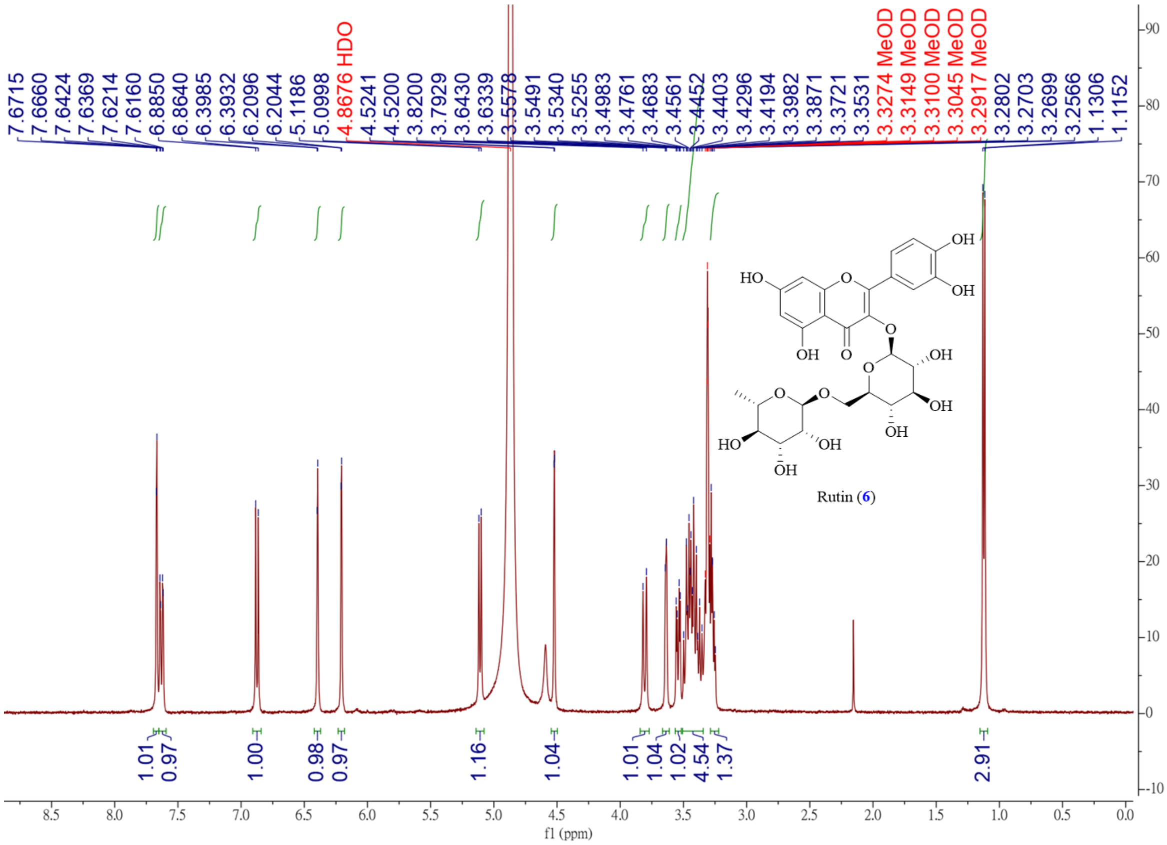

Supplementary Fig. 11. The ^{1}H-NMR spectrum (400 MHz, methanol-d_4) of rutin (**6**).

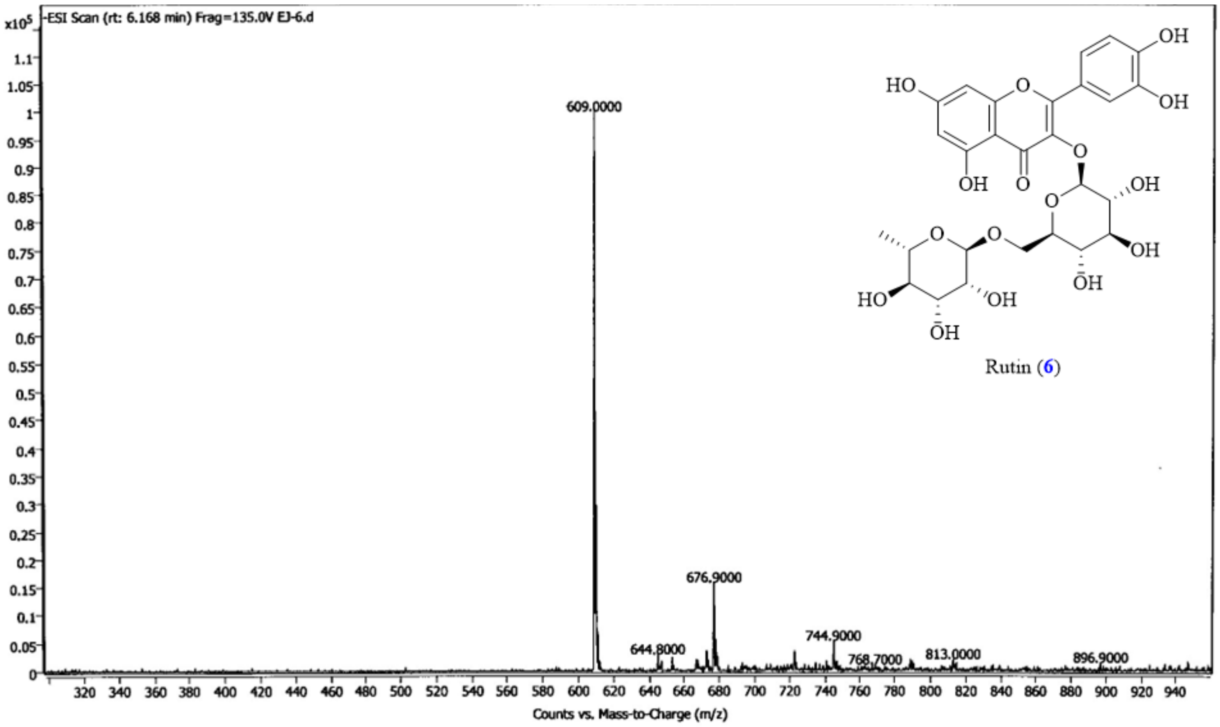

Supplementary Fig. 12. The ESI-MS spectrum of rutin (**6**).

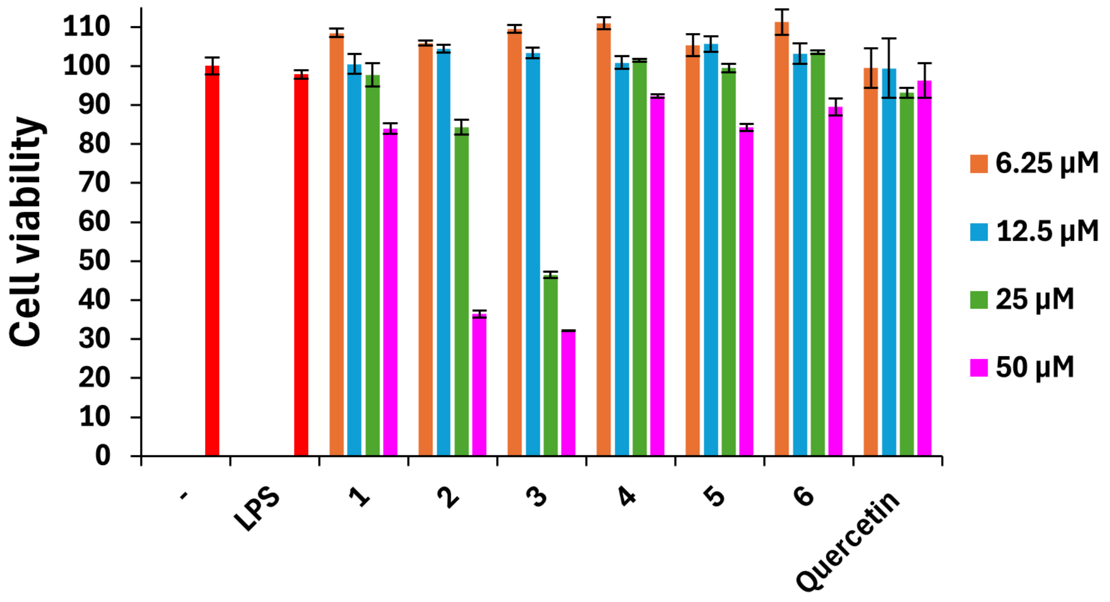

Supplementary Fig. 13. Cell viability evaluation of isolated components on RAW264.7 cells.
